# From words to action? Linking ESG reports to environmental performance

**DOI:** 10.1371/journal.pone.0350762

**Published:** 2026-06-16

**Authors:** Ivan Savin, Mateo López Carel, Eva Schlindwein

**Affiliations:** 1 ESCP Business School, Madrid, Spain; 2 Bern University of Applied Sciences, Bern, Switzerland; Shanghai Ocean University, CHINA

## Abstract

Companies increasingly use ESG reports to communicate their environmental priorities, yet it remains unclear whether the issues they emphasise correspond to measurable improvements in environmental performance. Using computational linguistics, we analyse the full texts of 1,477 ESG reports from companies listed in the STOXX Europe 600 index, identifying 34 main topics—six of which are related to environment. We describe how these topics evolve over time and differ across sectors, cluster companies based on their thematic focus, and test whether specific topics are associated with tangible low-carbon transition efforts. Our findings show that the alignment between environmental communication and environmental performance is topic-dependent. Topics such as sustainable value chains and renewable energy are associated with improvements in environmental KPIs, whereas others, including emissions or electric vehicles, show no significant association. Methodologically, our study advances prior work by combining structural topic modelling with firm-level KPI analysis to evaluate ESG disclosure at the level of specific environmental themes rather than aggregate reporting measures. Our study demonstrates how topic-specific reporting patterns relate to environmental outcomes and offers a more nuanced understanding of when ESG reporting reflects substantive environmental action.

## 1. Introduction

Companies across the globe now publish extensive ESG reports to explain their environmental commitments, climate strategies, and progress towards net-zero goals. These reports shape investment decisions, regulatory attention, and public evaluation of corporate responsibility. Yet a central problem persists: it remains unclear whether the environmental issues companies emphasise in their ESG reports correspond to actual improvements in environmental performance.

Prior research documents substantial gaps between “words” and “action,” in which firms communicate sustainability ambitions without equivalent changes in outcomes [1,2]. Concerns about greenwashing, selective disclosure, and unaudited information further undermine the credibility of ESG communication [3,4]. At the same time, ESG ratings differ widely across providers, in part because of inconsistencies in how firms report environmental information [5]. As a result, stakeholders cannot easily determine when companies move from words to action, and when ESG reporting remains primarily symbolic.

To address this gap, we examine two questions: What environmental topics do companies emphasise in their ESG reports, studying how these topics vary across countries, sectors and over time? To what extent are these environmental topics associated with changes in firms’ environmental key performance indicators (KPIs)?

To answer these questions, we apply structural topic modelling (STM) to 1,477 ESG reports from companies listed in the STOXX Europe 600 index, covering the period between 2010 and 2023. We identify 34 distinct topics and link the prevalence of these topics to changes in companies’ environmental KPIs - such as CO₂ emissions, energy use, and environmental innovation – over time and across sectors.

Our findings show that the environmental issues companies emphasise in their ESG reports vary widely in how they relate to corporate environmental performance. Topics related to sustainable value chains and renewable energy are associated with improvements in environmental KPIs, consistent with prior work linking specific forms of environmental communication to substantive behaviour [6]. In contrast, frequent discussion of emissions or electric vehicles shows no significant association with environmental performance, echoing concerns that some ESG reporting may function more as symbolic or compliance-driven communication [7]. These results indicate that ESG reporting contains both substantive and symbolic elements, and that the specific topics companies choose to emphasise can provide signals –though not guarantees– of their environmental actions.

This study makes two contributions. First, we show how the *content* of ESG reporting relates to firms’ environmental performance. Existing research has examined the volume, sentiment, or general presence of sustainability disclosures, but has not been able to assess whether the *specific environmental issues* firms emphasise correspond to measurable changes in environmental outcomes. By analysing the themes highlighted in ESG reports and linking them to environmental KPIs, we reveal which issues are more likely to reflect substantive environmental action and which do not.

Second, we demonstrate that the words–action gap is not uniform. Prior work typically treats discrepancies between sustainability communication and environmental performance as a firm-level condition. Our analysis shows that these discrepancies vary systematically by *topic*: some environmental themes are associated with improvements in environmental KPIs, whereas others show no relationship. This shifts the understanding of the words–action gap from a binary firm-level phenomenon to a patterned, content-dependent one, offering a more granular and diagnostic perspective on when ESG reporting aligns with action. By moving the analysis from aggregate ESG disclosure measures to the level of specific environmental themes, our approach provides a more precise diagnostic of when sustainability communication corresponds to measurable environmental action.

While prior computational studies have mapped ESG themes, sentiment, or pillar-level classifications, they typically focus on describing disclosure patterns or linking aggregate ESG indicators to financial outcomes. Our study differs in two respects. Methodologically, we link unsupervised structural topic modelling directly to firm-level environmental KPIs at the level of specific environmental themes. Substantively, we show that the relationship between ESG communication and environmental performance is topic dependent: some environmental themes are associated with measurable improvements, whereas others are not. This provides a more granular perspective on the word-action gap in ESG reporting.

The remainder of the paper is structured as follows. Section 2 reviews research across ESG reporting, greenwashing, environmental regulation, and environmental performance. Section 3 describes the data and the methodology employed, including STM and the KPI regression framework. In Section 4 we present the results on topic prevalence, sectoral patterns, firm clustering, and statistical associations between environmental topics and KPIs. Section 5 discusses implications for research and practice, and concludes.

## 2. Literature review

Research on ESG reporting, greenwashing, regulation, and environmental performance highlights long-standing challenges in evaluating whether companies’ sustainability communication reflects substantive environmental action. This section summarises the main insights from this work and identifies key limitations that motivate our focus on the environmental topics firms emphasise in their ESG reports.

### 2.1 ESG reporting and variation in environmental communication

ESG reports have become a primary channel through which firms communicate their environmental commitments, risks, and strategies. However, reporting practices differ markedly across firms and sectors. Companies vary in the level of detail they provide, the structure of their reports, and the environmental issues they prioritise [2,3]. Recent systematic reviews confirm that even under established standards, reporting practices vary widely in structure and emphasis, suggesting that firms retain considerable discretion over their environmental communication [4]. Empirical studies also find that topic emphasis differs substantially across countries and industries, reinforcing the idea that ESG reporting remains far from standardised [5].

Concerns about credibility have intensified as ESG reporting has expanded. Much ESG information remains unaudited, and recent analyses show that the relationship between disclosed policies and underlying environmental performance is often weak [6]. Persistent divergence in ESG ratings further reflects methodological inconsistencies in scope, measurement choices, and aggregation rules [7]. Recent work concludes that these divergences have structural origins and are unlikely to fully converge without substantive changes to data quality and verification processes [8]. Recent financial research also shows that environmental signals embedded in ESG disclosures can affect market risk perceptions by demonstrating, for instance, that firms perceived as environmentally friendly influence sectoral crash risk asymmetries, suggesting that environmental characteristics embedded in ESG communication can shape investors’ risk assessments [9].

Although this research documents substantial variation and credibility concerns, it offers little insight into whether the *specific environmental issues firms choose to emphasise* correspond to measurable improvements in environmental KPIs. Understanding this link matters because stakeholders often rely on the content of ESG reports to evaluate whether firms are addressing environmental priorities in a meaningful way.

### 2.2 Greenwashing and the persistence of the word–action gap

A central theme in the literature is the gap between firms’ sustainability communication and their environmental behaviour. Research documents numerous mechanisms of greenwashing, including selective disclosure, focus on positive achievements, and omission of negative outcomes [10,11]. Recent reviews indicate that research on greenwashing has expanded rapidly, with growing attention to symbolic disclosure strategies and impression management [12]. Modelling work further demonstrates that symbolic communication can be a rational choice for firms facing pressure to appear responsible in contexts with limited verification [13].

Textual analyses reveal that some reporting practices, such as vague statements, repeated boilerplate content (i.e., keywords and stable common phrases that can be applied in several contexts without needing significant modifications), or broadly framed environmental claims, may signal symbolic rather than substantive communication [14–18]. These findings suggest that ESG reports often contain a mix of substantive and symbolic elements. However, such analyses typically evaluate credibility or symbolic tendencies at the firm level, treating the word–action gap as a general characteristic of a company’s reporting.

These insights help explain why the word–action gap persists but do not reveal whether discrepancies differ by environmental issue. Some topics may entail more measurable commitments and thus be more closely tied to performance, whereas others may be easier to discuss symbolically. Identifying whether the alignment between word and action varies by environmental topic would allow for a more precise understanding of the credibility of ESG reporting.

### 2.3 Regulation and growing expectations for meaningful disclosure

Regulatory initiatives such as the EU’s Corporate Sustainability Reporting Directive (CSRD) and Sustainable Finance Disclosure Regulation (SFDR) seek to improve the reliability and comparability of ESG reporting [19–21]. Similar developments in the United States and other regions reflect growing expectations that enhanced reporting will provide stakeholders with more decision-useful information on environmental risks and performance [22–24].

Although these frameworks require firms to disclose more structured and detailed information, companies still retain substantial flexibility in selecting which environmental issues to highlight. Disclosure requirements often mandate reporting on strategies, risks, and governance structures but do not directly verify whether the issues emphasised in reports correspond to real changes in environmental KPIs [25–27]. This leaves considerable room for variation in how firms frame their environmental efforts.

This evolving regulatory landscape highlights the need for evidence on whether the *content* of ESG disclosures relates to environmental performance. Such evidence is essential for assessing how firms respond to new reporting requirements and for evaluating whether regulatory reforms improve the environmental transparency of ESG reporting.

### 2.4 Analysing ESG reporting content through topic modelling

Among most frequent machine learning (ML) methods for ESG reports in the literature is the so-called Topic Modelling (TM) methodology. Different TM algorithms have been employed to analyse ESG reports, mostly Latent Dirichlet Allocation (LDA) and its variations [28,29]. Another approach is taken by Schimanski et al. [30] who used supervised ML models, RoBERTa and DistilRoBERTa, to determine whether a sentence relates to any of the three pillars of ESG. Similarly, the study by Funk et al. [31] classifies ESG reports from companies in the STOXX Europe 50 to more specific categories within the three ESG pillars. Recent methodological reviews highlight the increasing use of distribution-sensitive econometric techniques to capture heterogeneous economic relationships, including quantile regression approaches that analyse effects across different parts of a distribution rather than only mean outcomes [32].

Yet, as a pretrained model, its capacity to provide new topics or trends that might emerge in the ESG domain in the future is limited. Furthermore, it possesses solely the ability to categorise the specific pillar of ESG reporting to which it pertains, yet it lacks the capacity to delve further into that classification.

The key methodological difference between topic modelling and the two studies above-mentioned is that TM performs an unsupervised classification, i.e., one does not have a given list of terms or sentences to be searched for by the algorithm. Dyer et al. [8] study the trends in 10-K reports during the period of 1996–2013 in the United States employing LDA finding that the presence of boilerplates and redundant texts in the documents has increased substantially during the studied period.

Yang and Yang [6] study the correlation between the information present in the ESG reports from 97 companies from the Fortune 500 during the 2010s and their financial key performance indicators like the ROA or Tobin’s Q. The authors find that companies addressing customer-oriented capabilities in their ESG disclosures are associated with better financial outcomes, which collaborates to the literature perception that ESG efforts being directly related with market value and profitability.

Baier et al. [33] focus on 10-K reports published by US companies. The sample is constituted by the documents published by the 25 largest companies in the S&P100 index during 2015–2019. These documents contain not only non-financial information but rather the global activities executed by a company throughout the year. The authors use LDA to reveal trends in these disclosures. Across all the reports included, the share of ESG-related words represented 3.7% of the total. The company with the lowest presence was Berkshire Hathaway (specialised in financial services) and the one which presented the highest proportion of ESG words was Johnson&Johnson.

Bowen and Min [34] study over 4500 articles from national newspapers and 456 academic papers related to ESG, published between 2012 and 2022 using LDA. For academic papers, they found a prevalence of topics related to what they labelled “Environmental Protection”, followed by “Sustainability” and “Financial Performance”. The key difference with the non-academic articles is the dominance of the topic “Carbon Emissions and Climate Neutrality” in newspapers.

Yet, across these contributions, one key shortcoming persists: a lack of evidence connecting ESG communication themes with companies’ real-world environmental performance. This disconnect limits our ability to distinguish between substantive ESG engagement and symbolic disclosure. The present study addresses this by directly linking ESG report content to changes in firms’ environmental KPIs.

## 3. Materials and methods

### 3.1 Data

The present study focuses on the ESG reports released between 2010 and 2023 by companies listed in the STOXX Europe 600 index. We concentrate on Europe for two reasons. First, the EU is one of the global leaders in decarbonizing its economy with many policies stimulating companies to reduce their emissions, such as the European carbon market (the EU-ETS). Second, it is one of the largest stock markets in the world covering companies from many countries and industries ensuring that the dataset will contain a diverse sample of companies.

We collected the ESG reports from the Sustainability Reporting Navigator (https://www.sustainabilityreportingnavigator.com/). At the time of data collection (8th of August 2024), the database encompassed a total of over 11,409 documents from 787 companies across 18 European countries not all of which listed in the STOXX Europe 600 index. It included five types of publications:

- Annual reports that provide financial information about the firm and general managerial decisions (in total, n = 6306 documents)
- ESG reports that are the ones which highlight the companies’ efforts in terms of ESG performance (n = 4056)
- Integrated reports that combine the above-mentioned two types (n = 248)
- Carbon disclosure project reports which are documents that are more specific to carbon emissions related efforts (n = 19)
- Other reports published by the organisations (n = 780).

We preselect only ESG reports to avoid more general reports the companies publish primarily on their financial situation. Full texts of 1489 reports that match companies from the STOXX Europe 600 were retrieved in a PDF format, which we subsequently digitalised. We successfully transformed 1477 documents while 12 files were corrupted (i.e., one could not extract text from the PDF) and had to be dismissed. In doing that we extracted only the textual data from the reports and omitted tables and graphs which is in line with the previous literature [25,35]. We did not limit the sample to a specific type of ESG reporting (e.g., GRI-based), as there is currently no consensus in the literature on which reporting format is superior in quality or reliability. Additionally, narrowing the scope to a particular standard would have significantly reduced the size and diversity of our dataset. Instead, we aimed to retain a heterogeneous sample that includes companies exhibiting different dynamics on environmental KPIs, allowing us to examine how the content of ESG reports is associated with actual performance. See the number of reports per country and per year in Table A1 in the Appendix (S1 File).

The ESG reports released are mostly written in English, but a few are in German, French or Italian. To ensure comparability of the textual data across the reports, we translated the non-English reports using the DeepL translator service. Translating documents into a common language prior to topic modelling is standard practice in cross-national computational text analysis, as it ensures corpus comparability while avoiding sample bias from excluding non-English documents [36]. Topic modelling relies on word co-occurrence structures rather than stylistic features, and prior research has shown that automatic translation tools provide reliable and statistically comparable results for text-as-data applications [37–39].

**Fig 1:**
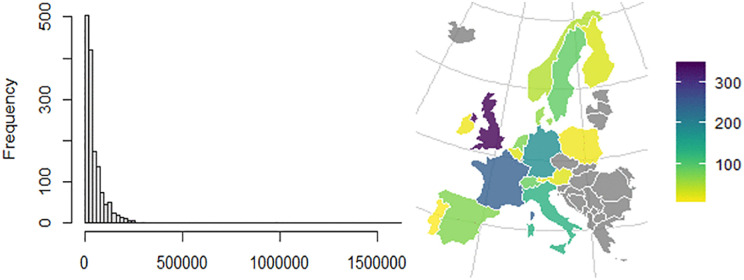
Distribution of textual documents in terms of length (left chart) and across countries (right chart).

The final dataset encompasses 1477 reports from 268 distinct companies from STOXX Europe 600. The average length of reports is 47,528 words, with a minimum of 411 and a maximum of 1,616,575 (left chart in Fig 1). The dataset covers 16 countries with the UK, France and Germany leading by a big margin (see right chart in Fig 1).

Furthermore, it is worth stressing that the final dataset is an unbalanced panel: while some companies are observed for all 14 years (for instance, Endesa, Infroma and Nokia), for some other firms we only have 1–2 reports (e.g., Ryanair and Bouyges). Additionally, for all companies in the dataset we obtained information on several environmental KPIs from the Refinitiv database such as *CO*_*2*_ *emissions, energy use, ESG score, emissions score, environmental innovation score.* This KPI data, however, is only available from 2019 to 2023. Several of these measures (like CO_2_ emissions and energy use) we subsequently normalise in relation to revenue to account for company’s size thus obtaining emission and energy intensity of firm output (see Table A2 in the Appendix (S1 File)).

**Fig 2:**
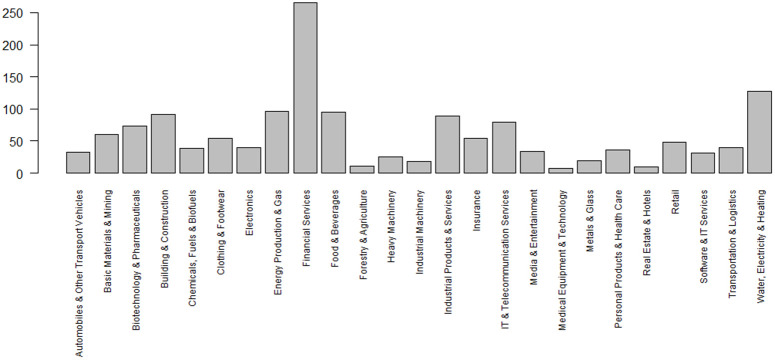
Number of reports per sector.

The firms in our dataset belong to different industrial sectors (see Fig 2). To reduce the number of sectors and avoid any sector having too few observations (like Forestry & Agriculture covering less than 1% of observations, see Table A3 in the Appendix (S1 File)) we group these sectors into seven sector groups as explained in Table 1 below. The primary aim of this grouping is to put together firms similar in their core business activity, like producers of transport vehicles and companies exploiting those vehicles to provide services (e.g., logistic services).

**Table 1:** Sector clusters and their prevalence in the dataset.

Sector group	Sectors	% of reports
Finance	“Financial Services”	21.60%
	“Insurance”	
Housing	“Real Estate & Hotels”	15.60%
	“Water, Electricity & Heating”	
	“Building & Construction”	
Digital	“IT & Telecommunication Services”	12.50%
	“Software & IT Services”	
	“Media & Entertainment”	
	“Electronics”	
Industrial	“Automobiles & Other Transport Vehicles”	7.90%
	“Industrial Goods & Services”	
	“Heavy Machinery”	
	“Industrial Machinery”	
	“Transportation & Logistics”	
Pharma	“Medical Equipment & Technology”	7.90%
	“Biotechnology & Pharmaceuticals”	
	“Personal Products & Health Care”	
Consumer Goods	“Retail,”	6.90%
	“Food & Beverage”	
	“Personal & Household Goods”	
	“Clothing & Footwear”	
Resources	“Basic Materials & Mining”	6.10%
	“Energy Production & Gas, Fuels & Biofuels”	
	“Metals & Glass”	
	“Forestry & Agriculture”	

**Fig 3:**
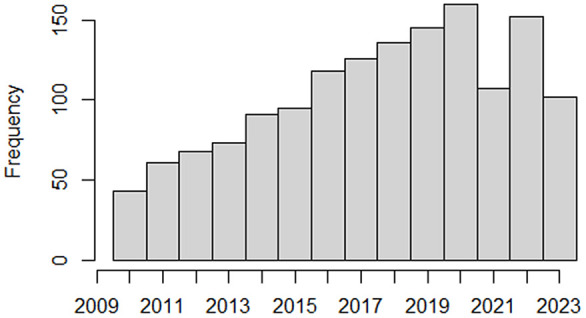
Number of documents per year included in the dataset.

Plotting the number of reports over time in the final dataset we see a significant upward trend in the number of reports published peaking in 2020 (Fig 3). In 2021 the number of reports drops which may be due to COVID-19 pandemic. Another drop in 2023 may be because not all reports for this year being collected by the Sustainability Reporting Navigator by the time we accessed the data.

This study relied exclusively on publicly available corporate ESG reports and secondary firm-level environmental KPI data. No human participants, personal data, animal subjects, surveys, or experiments were involved. Therefore, ethics committee approval and informed consent were not required.

### 3.2 Methods

To uncover latent topics from the ESG reports, we use the Topic Modelling (TM) approach. The main advantage of this approach compared to keyword search is that it allows words to change their meaning dependent on the context they are presented in [40]. TM clusters words in topics based on how often those words co-occur across different documents in our sample of ESG reports [41]. For example, if the words “emissions”, “carbon”, “factory”, “production”, “gas”, and “GHG” appear in a topic labelled “GHG emissions” (see Section 4), we can conclude that the topic is about GHG emissions.

The main advantage of TM is the ability to quickly process very large data sets that would be very costly to manage using manual coding [42]. Another advantage is that the classification of topics is not based on the programmer’s own judgment, but is objective in the sense it is based on the textual data of the ESG reports, and this approach is transparent and reproducible [33,43,44]. More specifically, we use the Structural Topic Modelling (STM) method. The advantage of STM compared to LDA, is that it can use metadata about the underlying texts (in our case, the sector group of the company the ESG report corresponds to and the year of publication). Thanks to this STM is proved to generate semantically more coherent (i.e., easier to interpret) and interpretable topics [45]. This further allows us to formally study how these themes differ across sectors and evolve over time, within one coherent modelling framework. The STM algorithm we used in this study is based on the corresponding package in R by [46].

Before analyzing the ESG reports with STM we had to pre-process the textual data. We transformed all capital letters to the lower case, deleted non-Latin characters, stop words (such as “we”, “and” or “like”) and words being shorter than three letters. Afterwards, all terms included in the dataset were lemmatised transforming words to their dictionary form. Words that appeared in less than three reports were eliminated, and stable two-word sequences called bigrams were additionally formed (e.g., “corporate_governance”, “ghg_emissions”, “hazardous_waste”). As a result, the final dataset contains 17,270 unique words and 6,727,732 total word occurrences for building a topic model. All these preprocessing steps follow standard practice for bag-of-words topic models and are appropriate for STM, which relies solely on textual information (e.g., 8,28,29,33). In the STM literature, such steps are treated as necessary normalization procedures rather than robustness dimensions to be systematically varied. Therefore, removing non-textual elements and applying lemmatisation or stop-word filtering does not meaningfully reduce the information available to the model [46,47].

Subsequently, despite individual documents often being very long, the total number documents (only 1477) is rather limited, particularly given the size of the data in terms of number of unique terms. Since STM, like any other TM method, attributes words to topics based on how often these words co-occur in the same textual documents, running STM on the 1477 documents considering each of them as unique observations leads to rather vague topics that are hard to interpret. To address this challenge, we follow Savin and et al [48] by splitting 1477 ESG reports into shorter texts of no more than 10000 words each, resulting in a total of 4709 separate texts Following this approach, and consistent with common practices in computational linguistics (e.g., [49]), the 10,000-word threshold increases the number of observational units while still capturing co-occurrences that may occur farther apart in substantially longer ESG reports and prevents a few exceptionally long reports from dominating the analysis. Topic prevalences were then aggregated back to the original report level using segment-length weights, such that each segment contributes proportionally to its word count in the original document, thereby reconstructing the report-level topic distribution, mirroring Savin et al. [50] and preserving the integrity of subsequent regressions. As a robustness check, we also tried a segmentation threshold of 3000 words, and the results remain robust (see Appendix B in S2 File).

To choose the appropriate number of topics, we follow the earlier literature [46,51,52] and compare STM models with three to 50 topics on such criteria as out-of-sample prediction accuracy (held-out log-likelihood), the extent to which popular words from topics overlap (exclusivity), and the degree to which words from the same topic appear in the same texts (semantic coherence). Fig A1 summarises the results indicating that 34 topics exhibit the maximum prediction accuracy and close to maximum exclusivity, while maintaining a relatively high semantic coherence (S1 File).

Preprocessing steps such as stop-word removal, lemmatization, and bigram construction follow standard practice in bag-of-words topic modelling and are intended to enhance interpretability rather than to define alternative model specifications [40,45,53]. Topic-number selection was based on a comparison of models ranging from 3 to 50 topics using held-out likelihood, semantic coherence, and exclusivity, consistent with established STM guidance (Roberts et al., 2019).

Once the topics are formed and their prevalence are estimated, we proceed by analysing factors explaining the variation of topic prevalence among the ESG reports. To this end, we regress topic proportions over the covariate variables we used in building our topic model [50]. In particular, a linear regression model was specified for each of the 34 topics (indexed by k) containing year of report publication and dummy variables for each of the sector groups as follows:


$$\begin{array}{cc} & {Topic\;Prevalence}_{k}\sim \;{Constant}_{k}+\;Year\;of\;ESG\;report+\;Finance\;+\;ConsumerGoods\;+\;Digital\;+\;Resources\\ & \;\;+\;Industrial\;+\;Housing\;+\;Pharma+\;{Residual}_{k}\; \end{array}$$ (1)

Results of these regressions on the whole data sample covering 1477 reports are then reported to demonstrate the distribution of topics over time and across sector groups.

**Fig 4:**
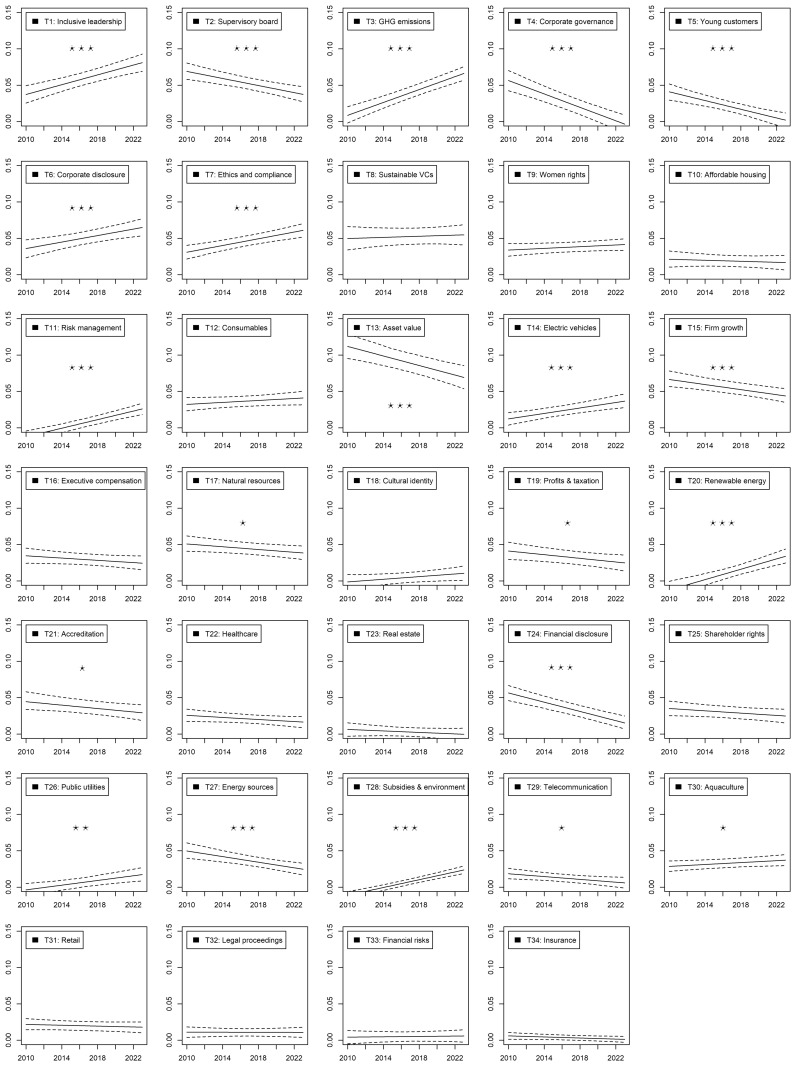
Trends in topic popularity over time. *Note*: Values generated by a regression where the outcome variable is topic prevalence and year of publication is the predictor. Confidence intervals plotted as dashed lines indicate the 95% uncertainty range and include both regression and measurement uncertainties associated with the STM model. ***, ** and * denote 0.1%. 1%, and 5%, significance, respectively.

Next, we cluster companies in terms of the environmental topics addressed, i.e., we group them based on prevalence of these topics in their ESG reports using hierarchical clustering. We focus on the topics related to environment (see Table 3) and limit the analysis to the period between 2019 and 2023. The reasons for selecting this timeframe are as follows. First, the dataset in unbalanced with only few companies covered in early years, and some of them not observed in the later years. Since topic prevalences changed considerably over time (see Fig 4 and Fig A2 in S1 File), comparing those companies with firms observed only in the later years would be misleading. Second, the number of reports published during these years is significantly higher compared to earlier periods (see Fig 3). Hence, for the clustering we consider only firms which published at least three reports 2 during this period to ensure the comparison is more balanced as 1–2 reports filed in the earlier or later years may still bias our conclusions. The resulting sample includes 125 companies covered in the period 2019–2023.

**Table 3:** Aggregation of 34 ESG reports topics into six thematic groups.

Thematic group	Prevalence (%)	Constituent topics	Prevalence (%)	N of ESG reports
G1: Organisation governance	33.22%	T1: Inclusive leadership	8.47%	133
		T2: Supervisory board	6.73%	92
		T4: Corporate governance	5.66%	94
		T6: Corporate disclosure	5.65%	49
		T15: Firm growth	2.81%	24
		T16: Executive compensation	2.68%	20
		T25: Shareholder rights	1.22%	6
G2: Environment	18.48%	T3: GHG emissions	6.62%	65
		T8: Sustainable VCs	4.90%	119
		T14: Electric vehicles	3.19%	57
		T20: Renewable energy	1.81%	42
		T27: Energy sources	1.00%	12
		T28: Subsidies & environment	0.96%	12
G3: Sector-specific issues	15.78%	T10: Affordable housing	4.24%	65
		T12: Consumables	3.72%	88
		T17: Natural resources	2.28%	60
		T22: Healthcare	1.49%	27
		T23: Real estate	1.42%	29
		T26: Public utilities	1.00%	19
		T29: Telecommunication	0.85%	10
		T30: Aquaculture	0.78%	20
G4: People and community	13.07%	T5: Young customers	5.65%	89
		T9: Women rights	4.70%	20
		T18: Cultural identity	2.09%	52
		T31: Retail	0.64%	12
G5: Financial aspects	11.61%	T11: Risk management	4.14%	67
		T13: Asset value	3.62%	72
		T19: Profits & taxation	1.97%	58
		T24: Financial disclosure	1.25%	28
		T33: Financial risks	0.39%	2
		T34: Insurance	0.24%	0
G6: Public regulation	7.84%	T7: Ethics and compliance	5.48%	17
		T21: Accreditation	1.77%	17
		T32: Legal proceedings	0.59%	0

After identifying topics addressed in ESG reports, their distribution over time and across sectors, and forming company clusters based on how (dis)similar they are in environmental topics addressed in their ESG reports, we study how prevalence of certain topics is associated with firms’ actual efforts for climate change mitigation captured by environmental KPIs. In doing that we distinguish between KPIs provided directly by the companies (such as amount of CO_2_ emissions and energy used in proportion to revenues generated) and the KPIs assigned by Thomspon Reuters/Refinitiv (like ESG score, emissions score, environmental innovation score). Given that KPI data are available only for 2019–2023, our analysis should be interpreted as evidence of contemporaneous alignment between reporting emphasis and environmental performance rather than long-term dynamics.

To this end, we perform a regression analysis putting change in each of the KPIs on the left-hand side of the equation and change in the prevalences of the six topics related to environment on the right-hand side. This is meant to capture whether companies increasing discussion of certain environmental topics tend to improve their KPIs. Moreover, next to the change we also add the level of the prevalence of the topic since companies stressing environmental issues in their ESG reports in absolute terms may tend to improve their environmental KPIs more. All six environmental topics are included simultaneously, so coefficients represent conditional associations—i.e., relationships net of competing reporting themes—rather than isolated correlations.


$$\begin{array}{cc} & \Delta {KPI}_{i,t}\;\sim \;{T\text{3}\;GHG\;emissions}_{i,t}+{T\text{8}\;Sustainable\;VCs}_{i,t}+{T\text{1}\text{4}\;Electric\;vehicles}_{i,t}+{T\text{2}\text{0}\;Renewable\;energ}_{i,t}\\ & \;+{T\text{2}\text{7}\;Energy\;sources}_{i,t}+{T\text{2}\text{8}\;Subsidies\;\&\;environment}_{i,t}+\;{\Delta T\text{3}\;GHG\;emissions}_{i,t}\\ & \;+{\Delta T\text{8}\;Sustainable\;VCs}_{i,t}+{\Delta T\text{1}\text{4}\;Electric\;vehicles}_{i,t}+{\Delta T\text{2}\text{0}\;Renewable\;energ}_{i,t}+{\Delta T\text{2}\text{7}\;Energy\;sources}_{i,t}\\ & \;+{\Delta T\text{2}\text{8}\;Subsidies\;\&\;environment}_{i,t}+\;\beta_{t}+\;{\gamma_{i}+Residual}_{k}\; \end{array}$$ (2)

In addition, we add year ($\beta_{t}$) and company ($\gamma_{i}$) fixed effects (see Equation 2). Inclusion of the company and year fixed effects is meant to capture idiosyncratic differences between companies (e.g., smaller firm in the digital sector producing less emissions than a large company in the energy sector) and changes in the KPIs specific for a particular year (e.g., COVID-19 pandemic). Inclusion of these fixed effects also captures such stable company characteristics like size, age and sector. We also apply Driscoll and Kraay standard errors [54] that account for heteroskedasticity serial correlation and cross-sectional dependence.

Including both the level and the change of topic prevalence follows the logic used in empirical studies such as Dosi et al. [55], who show that levels and changes of an explanatory variable capture different mechanisms: levels reflect persistent, structural heterogeneity across firms, while changes capture dynamic adjustments that are more directly linked to performance outcomes. Put differently, the level captures a firm’s stable, long-run emphasis on a given environmental theme, whereas the change captures short-term adjustments in reporting focus, enabling us to separately identify structural heterogeneity and dynamic effects. In our context, the level of a topic indicates a company’s stable emphasis on an environmental theme, whereas the change reflects year-to-year shifts in disclosure. Omitting either would force us to ignore one of these channels or absorb it into fixed effects. Including both is therefore appropriate, even if non-standard, because it allows us to distinguish persistent disclosure strategies from dynamic adjustments in ESG reporting—exactly the distinction that motivated the dual specification in Dosi et al. [55].

To ensure that including both level and change terms does not introduce multicollinearity concerns, we computed variance inflation factors (VIFs). All VIF values are within the interval between 1 and 3 which well below the conventional threshold of 5, indicating that collinearity does not materially affect the estimates.

Two main hypotheses associated with Equation 2 that we are testing are:

H1: Companies with higher share of environmental topics in their ESG reports are expected to improve their environmental KPIs.

H2: Companies with increasing share of environmental topics in their ESG reports are expected to improve their environmental KPIs.

The corresponding null hypotheses are implying that there are now such associations. The regression analysis described in Equation (2) is used to empirically test H1 and H2 against these null hypotheses.

The motivation for the abovementioned hypotheses is the expectation that companies stressing certain environmental topics in their ESG reports reflect their actual efforts in improving their performance in this area [6]. Alternatively, if empirical data will not be able to provide any evidence in support of the hypotheses, we may conclude that companies’ discussion of environmental issues in their ESG reports may be interpreted as a sign of greenwashing.

## 4. Results

### 4.1 Main topics

34 main topics (denoted by the prefix “T”) we identified in the ESG reports are presented in Table 2 with their label, prevalence and most frequent and exclusive words. The largest topic is *Inclusive leadership* (T1) with the share of 8.47% of all the ESG reports. This topic is mostly related to various actions and activities enhancing employee performance and their well-being. This is in line with Sarkar and Searcy [56] and Goloshchapova et al. [28] who find *employee well-being* as one of the key subjects in ESG disclosures. The second most popular topic is *Supervisory board* (T2, 6.73%). This topic covers the communication around the different internal politics of the organisation, their main governance modifications and other matters that might affect the upper-level administration of the firm. Closely behind, the next most prevalent topic is *GHG emissions* (T3, 6.62%). This is the most prevalent topic related to environment, but not the only one.

**Table 2:** 34 main topics in ESG reports.

Topic	Prevalence (%)	Most frequent and exclusive words
T1: Inclusive leadership	8.47%	people – support – empower – leader – inclusive – diverse – wellbeing
T2: Supervisory board	6.73%	supervisory_board – executive – member – chair – nomination – committee – ceo
T3: GHG emissions	6.62%	emission – water – consumption – scope – ghg_emission – mwh – hazardous_waste
T4: Corporate governance	5.66%	director – committee – meeting – internal – statutory_auditor – appointment – event_discrepancy
T5: Young customers	5.65%	people – student – young – tomorrow – app – volunteer – entrepreneur
T6: Corporate disclosure	5.65%	information – reporting – stakeholder – data – topic – human_right – explanation – reporting
T7: Ethics and compliance	5.48%	compliance – audit – policy – internal – integrity – corruption – reputational
T8: Sustainable VCs	4.90%	sustainability – employee – work – standards – emissions – process – agreement
T9: Women rights	4.70%	employee – woman – hire – discrimination – female – workforce
T10: Affordable housing	4.24%	home – build – community – development – charity – modern slavery – landlord
T11: Risk management	4.14%	investment – risk – strategy – impact – lending – solvency – catastrophe
T12: Consumables	3.72%	product – food – supplier – packaging – plastic – palm_oil – forest
T13: Asset value	3.62%	asset – income – liability – value – goodwill – impairment – liability
T14: Electric vehicles	3.19%	vehicle – safety – battery – cobalt – automotive – semiconductor – engineer
T15: Firm growth	2.81%	growth – development – client – market – organic – productivity – revenue
T16: Executive compensation	2.68%	performance – compensation – bonus – salary – variable – incentive – termination
T17: Natural resources	2.28%	mine – metal – safety – steel – iron – refinery – copper
T18: Cultural identity	2.09%	initiative – activity – support – community – abroad – men – women
T19: Profits & taxation	1.97%	share – ordinary – profit – amortisation – taxation – scheme – misstatement
T20: Renewable energy	1.81%	project – action – development – photovoltaic – electrification – decarbonisation – hydroelectric -
T21: Accreditation	1.77%	accreditation – indicator – risk – activity – bureau_veritas – universal_registration – inspection
T22: Healthcare	1.49%	health – patient – treatment – research – pharmaceutical – drug –clinical_trial
T23: Real estate	1.42%	portfolio -rent – real_estate – asset – tenant – occupancy – vacancy
T24: Financial disclosure	1.25%	registration – information – consolidated – shareholder – fiscal – december
T25: Shareholder rights	1.22%	subscription – shareholder – delegation – right – general_meeting – authorize – holder
T26: Public utilities	1.00%	waste – plant – service – system – energy – heating – municipal – purification
T27: Energy sources	1.00%	gas – power – electricity – oil – nuclear – storage – coal
T28: Subsidies & environment	0.96%	taxonomy – scope – eligible – environmentally – capex – kpi
T29: Telecommunication	0.85%	cloud – software – mobile – technology – iot – analytics – connectivity
T30: Aquaculture	0.78%	fish – blue_revolution – seawater – farm – salmon -sealouse – biological
T31: Retail	0.64%	customer – service – help – overview – sale – income
T32: Legal proceedings	0.59%	court – appeal – lawsuit – tax – proceeding – defendant – claim
T33: Financial risks	0.39%	account – exposure – security – market – fair_value – default – collateral
T34: Insurance	0.24%	euro – capital – issue – insurance – corporation

To facilitate a more streamlined analysis of emerging trends, we group the topics in six thematic groups (designated with the prefix “G”, see Table 3), reporting also the number of ESG reports dominated by each topic*.* The latter allows to see that some topics can be dominant in many reports despite their not so big overall prevalence (e.g., T8 on sustainable VCs), while others are rather complementary despite occupying rather large prevalence overall (e.g., T6 on corporate disclosure). The first thematic group (G1) is *Organisation governance* which includes several topics that address management of the organisation and the stakeholders that do have direct decision power. It is by far the most prevalent thematic group (33.22%) and includes the two most frequent topics.

The second group by order of prevalence is the one that comprehends the discussion related to *Environment* (G2). It accounts for 18.48% in the ESG reports which demonstrates that it holds a major position in the list of priorities for companies in terms of non-financial aspects. This thematic group covers topics related to emissions (T3), value chains (T8), electric vehicles (T14), renewable energy (T20), energy sources (T27) and subsidies related to environmental KPIs (T28).

As the third thematic group (G3) we have a category labelled as *Sector-specific issues* corresponding to more specific issues that might be isolated from the rest of ESG subjects that appear because of the corresponding company belongs to a certain industry such as healthcare, real estate or telecommunications.

G4 encompasses topics related to the social pillar of ESG, with a strong focus on diversity, inclusion, and the equal treatment of minorities. It also addresses the relationship between companies and their clients, considering customer characteristics and specific needs. It covers 13.07% of the ESG reports. Thus, this area (S) results to be the last among the ESG pillars ranked by prevalence.

G5 includes predominantly financial topics, which continue to be reflected in ESG disclosures. Lastly, G6 covers topics related to companies’ interactions with public and regulatory institutions, focusing on regulatory compliance and accreditation.

With these results, the governance pillar (associated with G1, G5 and G6) gathers the most attention from organisations, since it encompasses the majority of topics and most of the associated textual data (52.08% total prevalence). Surpassing the prevalence of the two other pillars combined, this result is in accordance with the findings from Baier et al. [33] and Sarkar and Searcy [56].

### 4.2 Topic popularity over time

After obtaining the topic clusters and their prevalence, we proceed to study their evolution over the thirteen years period included in the dataset. The output is visualised in two figures, Fig 4 showing the statistical linear trends of the topics and their significance, and Fig A2 displaying the actual variation in topic prevalence over time (S1 File).

The most prevalent topic studied, Inclusive leadership, has experienced a significant upward trend from a 5% prevalence to 15%, being the most popular topic in ESG reports. In contrast, topics T2 and T5 on supervisory board and young customers lost popularity from around 10% to less than 5%.

Zooming on the topics related to the environmental pillar of ESG, T3 on GHG emissions is confirmed as a subject that has gained a lot of attention over the last 15 years, reaching its peak in 2021 (15%). In a similar manner, prevalence of Electric vehicles (T14) also increased across the period observed. The theme covers different characteristics, needs and possibilities of these vehicles. Correspondingly, T20’s (Renewable energies) prevalence distribution over time exhibits a significant upward direction. After hitting a low in 2016, its prevalence steadily increased. Prevalence of T28 on subsidies and environment also increased, particularly over the last five years. On the other hand, T27 on various energy sources (from coal, oil and gas to nuclear) has lost prevalence among companies’ sustainability disclosures, which reflects the shift in attention from fossil fuels to renewable energy (T20).

These tendencies align closely with the growing stringency of climate policies in the EU, but also with the introduction of the “double materiality” concept, This concept encourages organisations not only evaluate how climate and other risks and opportunities impact organisations, but also assess how companies’ actions impact the planet. The concept started to gain traction and was officially introduced in the corporate reporting landscape with the release of the EU Non-Financial Reporting Directive (NFRD) in 2014. However, it became widespread only in 2019 when the European Commission formally described the in the context of ESG reporting [57].

### 4.3 Topic concentration across sectors

**Fig 5:**
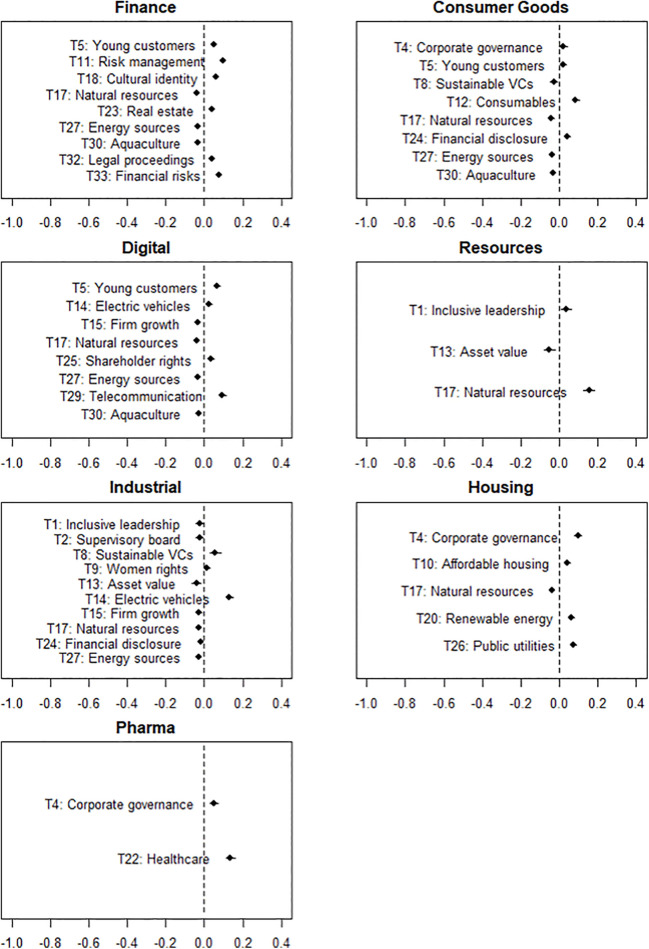
Topic concentration across sector clusters. *Note*: Only results significant at 5% level are reported. The plots show mean difference in topic proportions between the sector cluster under consideration and the rest of the sample (a positive value on the X-axis indicates a larger prevalence in the corresponding sector).

After identifying topics and their prevalence over time, we distinguish which topics are most associated with different sector groups that we formed earlier (see Table 1). Fig 5 visualises the topics with either a positive or negative significant association with the different sector groups. We obtain a significant positive association of T5 (Young customers), T11 (Risk management), T18 (Cultural identity), T23 (Real estate), T32 (Legal proceedings) and T33 (Financial risks) with the Finance sector. While T11, T23, T32 and T33 are logical topics that are easy to link with the industries related to finance, T5 and T18 emerge as unexpected subjects to have attired attention from companies. Contrarily, there is a negative significant association with T17 (Natural resources), T27 (Energy sources) and T30 (Aquaculture), which, again, appears to be logical based on the nature of the sector.

In the case of the Consumer goods sector, the analysis shows a strong positive association with T12 (Consumables) and T24 (Financial disclosure), as well as slight associations with T4 (Corporate governance) and T5 (Young customers). Conversely, this sector demonstrates negative associations with the same topics as the Finance sector (T17 and T27), with the addition of T8 (Sustainable value chains). While the majority of the associations align logically with the sector priorities, it is possible that T8 appears less than expected beforehand, what could suggest a need for greater emphasis on the role of global supply chains within these industries.

The Digital sector has a significant positive association with T5 as well (which may be explained by the role of young customers as main consumers) and other related topics such as T29 (Telecommunications) and T14 (Electric vehicles). T25 (Shareholder rights) is also strongly present in this sector. Contrary, T15 (Firm growth) as well as T17, T27 and T30, on average are stressed much less in ESG reports of companies in this sector.

T1 (Inclusive leadership) and T17 (Natural resources) tend to be stressed more in the Resources cluster, while for T13 (Asset value) the situation is the opposite. This presents an interesting tendency to social value over the financial one.

T14 is the most positively associated topic in the Industrial sector which can be explained by a large share of automotive companies in this sector. T8 also tends to be stressed by industrial firms more. The slight positive association with T9 (Women rights) deserves to be highlighted as it is one of the issues that have been brought back to attention of the STEM companies at several occasions because of the lack of female employees in the industry [58,59].

Housing sector is significantly positively associated with T4 (Corporate governance), T10 (Affordable housing) and T26 (Public utilities), which is logical as the issue is directly linked to these companies’ activities. T20 (Renewable energy) is also associated with the ESG reports published by companies in this sector, as the matters included in this topic are connected with the sector activities (think of solar photovoltaic panels installed on house roofs). The last sector cluster, Pharma, tends to stress stronger T4 (Corporate governance) as well as T22 (Healthcare).

### 4.4 Clustering firms based on topics related to environment

**Fig 6:**
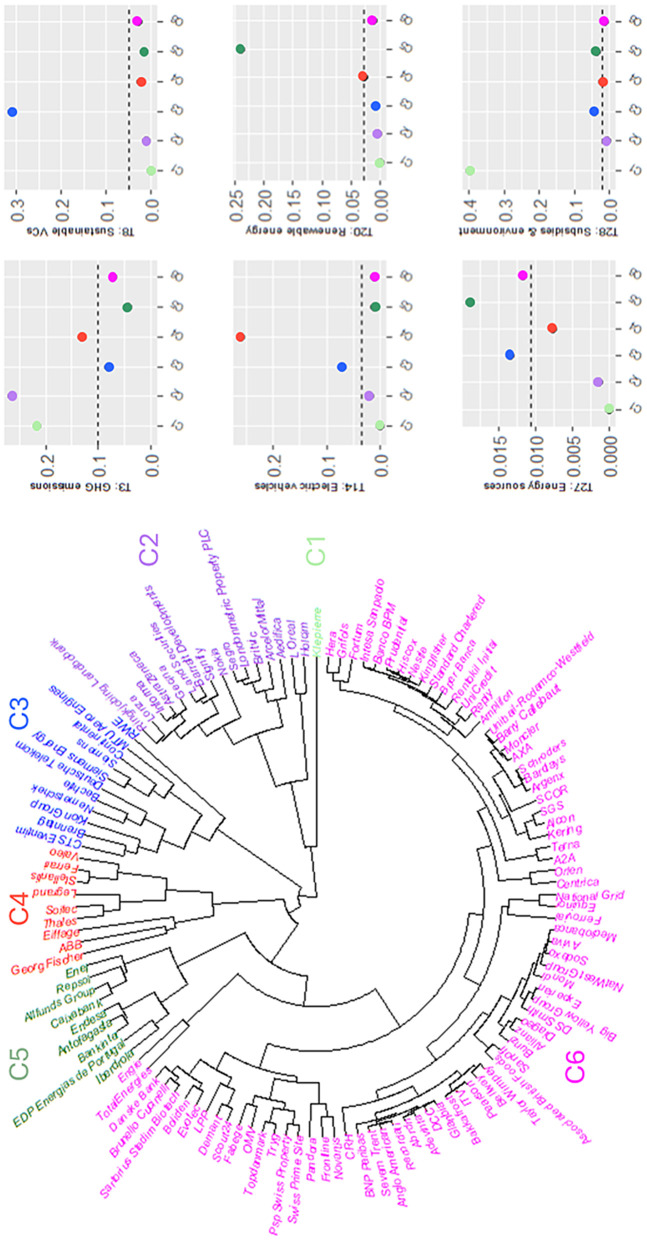
Six company clusters (left plot) and their focus on the topics on environment. *Note:* Left plot is a radial dendrogram clustering companies based on topic prevalence in their ESG reports. Right plot shows average prevalence of each of the six topics related to environment among the identified clusters of companies. Note that the y-axes of the six graphs have different scales. Dashed lines indicate the mean for each topic across the five clusters. Colours are consistent on the charts of the figure.

Estimating average prevalence for each of the six topics (T3, T8, T14, T20, T27 and T28) for each of the 125 companies in the sample for the period 2019−2023 and applying hierarchical clustering, we obtain the dendrogram reported in Fig A3 in Appendix (S1 File) and decide for 6 clusters highlighted with different colours. Left plot in Fig 6 presents this dendrogram in a radial form to enhance its readability. The six clusters (designated with the prefix “C”)*.* have the following sizes: C1 is an outlier cluster with a single company, C2 - 16, C3 – 11, C4 – 9, C5 - 9 and C6 (the largest cluster) includes 79 companies. Right plot in Fig 6 compares the clusters in terms of the six topics related to environment. The clustering exercise is exploratory in nature and complements the regression analysis by illustrating how firms group according to their thematic emphasis rather than establishing predictive or causal classifications.

The outlier cluster (C1) comprising the only company, Klepierre (French real estate firm), displays below-average prevalence of all topics except T28 on subsidies & environment (40%) and T3 on GHG emissions (over 20%). Exceptionally high focus on T28 makes this company stand out from the rest of the sample.

Cluster 2 (C2, including companies like L’Oreal) has a strong focus on GHG emissions (almost 35%), considerably above the average (10%). Contrarily, cluster 5 (including among others energy companies Enel and Repsol) does not emphasise this topic (only 5% prevalence).

Sustainable VCs (T8) are primarily addressed by 11 companies in cluster 3 (on average, prevalence is above 30%). Among these companies are industrial and energy companies like Siemens and RWE. On average, the topic covers 35% of the content of the 11 companies clustered in this group. The topic share among other four clusters is significantly lower (under 5%).

T14 on Electric vehicles tends to be stressed much stronger (on average, over 25%) among companies in cluster 4 which includes many companies specialised in automotives (like Ferrari) and transport services (e.g., Georg Fischer). Cluster 3 (which includes several manufacturers of transports components, such as Bechtle, MTU Aero Engines or Continental) also shows an above-average emphasis on the subject, although at a much lower level (around 7%).

T20 on renewable energy is predominantly present (average share of 25%) in ESG reports of companies in cluster 5, largely composed by energy companies (Enel, Repsol, EDP). It is worth noting that the share of this topic in other clusters is very low (below 5%). Finally, T27 on Energy sources logically is also stressed by cluster 5 the most.

The largest cluster (C6) shows a below-average prevalence for most of the environmental topics. The only exception is T27, stressing the role of different fossil and non-fossil fuels (e.g., nuclear), but not providing any concrete solutions on how to reduce emissions.

**Fig 7:**
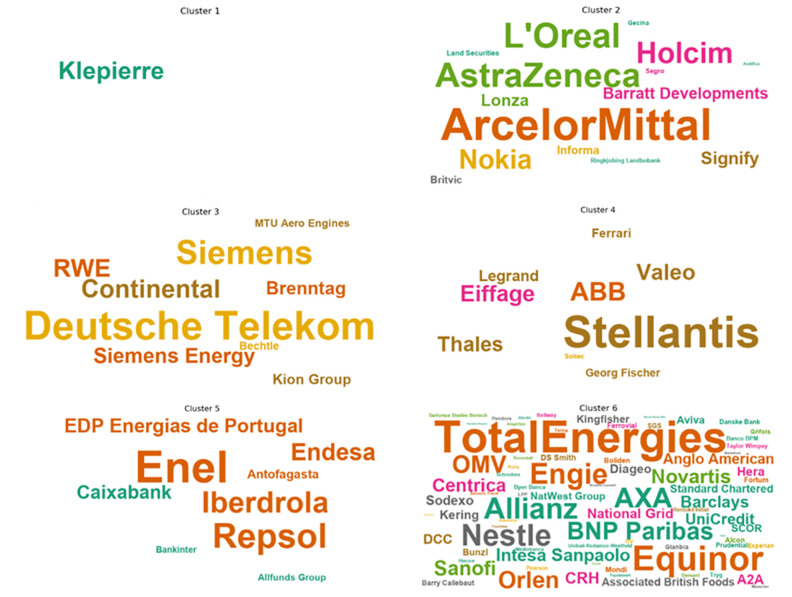
Company clusters based on prevalence of environmental topics. Note: Wordclouds showing the different company clusters based on similarity in prevalence of the environmental topics they address in ESG reports. Font size in a cloud reflects the revenue of the company in 2023, while colour indicates the sector: Finance, Resources, Housing, Pharma, Digital, Industrial and Consumer Goods. Companies can be compared by revenues from one cloud to the other.

Fig 7 demonstrates sector composition of the six clusters, while Fig A4 in Appendix (S1 File) their country composition. Clusters 2 (C2) and 6 are rather diverse both in terms of country and sector presence. C3, in contrast, is dominated by German companies from Digital and Resources. C4 is mainly concentrated in Industrial sector with companies predominantly from France and Switzerland. C5 is composed of companies in Resources, Housing and Finance based in Southern Europe (Spain, Italy and Portugal).

The identified clusters can be interpreted as reflecting distinct strategic orientations: for example, firms emphasizing renewable energy and energy sources (Cluster 5) are predominantly concentrated in energy-intensive sectors and appear more directly exposed to regulatory transition risks, whereas firms in Cluster 3 emphasizing sustainable VCa may reflect supply-chain restructuring strategies. In contrast, the largest cluster shows limited emphasis across environmental themes, suggesting a more compliance-oriented or less strategically differentiated disclosure approach.

### 4.5 Nexus between environmental topics and climate mitigation efforts

Table 4 provides results of the linear regression of environmental KPIs on topic prevalences including year and company fixed effects as explained in Equation 2 (Section 3). The first two columns report results for the indicators reported directly by the companies (emission intensity and energy intensity), while the latter three models analyse scores provided to the companies by ESG rating agency (in our case, Refinitiv). The relatively low R² values for some KPIs reflect the fact that year-to-year changes in environmental indicators are driven by many factors beyond ESG report content. Nevertheless, the significant coefficients indicate that specific topics still contain meaningful explanatory information even within this noisy and highly multifactorial setting.

**Table 4:** Regression of environmental KPIs on topic prevalences in ESG reports.

	Δ Emission intensity	Δ Energy intensity	Δ ESG score	Δ Emissions score	Δ Environmental innovation score
T3 GHG emissions	0.062 (0.231)	0.147 (0.212)	−0.027 (0.048)	0.024 (0.073)	−0.121 (0.203)
T8 Sustainable VCs	0.370 (0.704)	−0.791 (0.663)	0.126 (0.126)	−0.291 (0.242)	0.150 (0.278)
T14 Electric vehicles	1.224 (1.388)	0.089 (0.232)	−0.145 (0.131)	0.592 (0.390)	−0.050 (0.155)
T20 Renewable energy	−7.070^***^ (1.086)	−0.186 (0.937)	−0.041 (0.125)	0.033 (0.046)	−0.244 (0.242)
T27 Energy sources	64.016^***^ (13.645)	−2.273 (3.109)	−0.389 (0.408)	1.526 (1.630)	2.076 (1.832)
T28 Subsidies & env-t	2.342 (1.186)	0.815 (0.994)	−0.376^**^ (0.164)	−0.654^**^ (0.258)	−0.404 (0.266)
ΔT3 GHG emissions	0.0001 (0.0004)	0.0001 (0.0001)	0.0001 (0.0001)	−0.0001 (0.0001)	−0.0001 (0.0001)
ΔT8 Sustainable VCs	−0.006^***^ (0.001)	0.0001 (0.0002)	−0.0001 (0.0001)	−0.0002^**^ (0.0001)	−0.0001 (0.0001)
ΔT14 Electric vehicles	−0.0001 (0.0001)	0.0001 (0.0001)	−0.0001 (0.0001)	−0.0001 (0.0001)	0.0001 (0.0001)
ΔT20 Renewable energy	0.0001 (0.0001)	−0.0001 (0.0003)	−0.0001 (0.0001)	0.0001^**^ (0.00001)	0.001^***^ (0.00001)
ΔT27 Energy sources	−0.0003 (0.0004)	−0.0002 (0.0003)	0.0001 (0.0001)	−0.0002 (0.0002)	−0.0002 (0.0005)
ΔT28 Subsidies & env-t	−0.0004 (0.0005)	−0.0002 (0.0002)	0.0001 (0.0001)	0.002^***^ (0.0003)	0.0001 (0.001)
Year FEs	Yes	Yes	Yes	Yes	Yes
Company FEs	Yes	Yes	Yes	Yes	Yes
N obs	318	287	331	326	268
R^2^	0.584	0.022	0.046	0.163	0.183

**Note:** Robust standard errors are reported in parentheses. Asterisks ^***, **^ and ^*^ denote 0.1%, 1% and 5% significance, respectively. Shaded cells indicate statistically significant results. Different number of observations across regressions is due to missing information on the KPIs for certain companies.

Our results demonstrate that companies that increase discussion of sustainable value chains (ΔT8) tend to improve their performance both in self-reported measure of emission intensity and Emissions score from Refinitiv. This coefficient on ΔT8 thus captures a short-run effect: in years when a company increases the share of its ESG report devoted to sustainable value chains, it tends to experience a small but significant reduction in emission intensity (6 kg of CO2 per m USD) and an improvement in its emissions score (by 0.002 points), compared to years in which its reporting focus remains unchanged. Furthermore, more focus in ESG reports on renewable energy (T20) and less on a more general topic of energy sources (T27) is positively and significantly associated with reduction in CO_2_ emissions indicating that companies focusing on renewable and not fossil fuels are actually reducing their emissions. Here, the coefficients on the levels of T20 and T27 reflect long-run reporting orientations: firms that systematically devote a larger fraction of their ESG reports to renewable energy tend, on average, to achieve significant reductions in emission intensity (1 tone of CO2 per m USD for 1% higher share of the topic), whereas firms that place relatively more emphasis on general energy sources (including fossil fuels) tend to increase their emission levels.

Furthermore, we find that companies increasing their discussion of renewable energies in their ESG reports (ΔT20) tend to increase their performance in emissions and environmental innovation scores. This indicates a dynamic component: years in which a firm increases its emphasis on renewable energy (a higher ΔT20) are also years in which its emissions and environmental innovation scores improve, suggesting that shifts in reporting towards renewables coincide with incremental progress on these KPIs. The latter is also intuitive since renewable energy technologies are still relatively new compared to technologies based on fossil fuels and require considerable R&D investments for their implementation [60,61].

The topic 28 on subsidies and environment, in contrast, seems to demonstrate a mixed relationship with ESG and emissions scores of the companies. The negative coefficients on the level of T28 imply that firms whose ESG reports are systematically more focused on environmental subsidies and taxonomy-related issues tend to exhibit lower ESG and emissions scores, whereas the positive coefficient on ΔT28 shows that short-run increases in attention to this topic are associated with slight improvements in the emissions score, consistent with firms seeking external support while undertaking emission-reduction efforts. Topics T3 on GHG emissions and T14 on electric vehicles, do not have any significant statistical association with environmental KPIs. Taken together, the level coefficients capture how firms with a persistently stronger emphasis on particular environmental topics differ in their KPI trajectories, while the change coefficients (ΔT) capture how year-to-year shifts in reporting emphasis co-move with short-run KPI changes. The results allow us to conclude that the empirical evidence partially supports H1, as higher levels of certain environmental topics—most notably Renewable energy (T20)—are significantly associated with lower emission intensity, whereas others show no detectable relationship. Regarding H2, we find support for the idea that increases in discussion of specific topics (e.g., T8 on Sustainable VCs; T20 on Renewable energy) correspond to improvements in selected KPIs, particularly emissions-related scores. For the remaining topics, the estimated effects are statistically insignificant, meaning we fail to reject the null hypotheses. These results indicate that the hypotheses hold for some environmental themes but not universally across all topics.

Some coefficients in Table 4 exhibit signs that may appear counterintuitive, for example, the positive level coefficient of T8 (Sustainable VCs) for emission intensity. However, these estimates are statistically insignificant and therefore do not provide evidence of a systematic association. We refrain from interpreting such coefficients.

## 5. Discussion and conclusion

This study makes two contributions to research on ESG reporting and environmental performance by examining whether the environmental themes emphasised in ESG reports correspond to measurable improvements in firms’ environmental KPIs. First, we show that the *specific environmental topics* emphasised in ESG reports vary in whether they are associated with measurable improvements in environmental KPIs. Prior research has examined disclosure volume or sentiment [7,8], aggregated ESG indicators [5], or general communication–performance relationships, but has not assessed whether the content of ESG reporting—what firms actually choose to talk about—provides meaningful information about environmental outcomes. By linking topic-level disclosure to changes in environmental KPIs, our analysis offers a more granular understanding of when sustainability communication reflects substantive environmental action.

Second, we demonstrate that the word–action gap is not uniform across firms but varies systematically across environmental issues. Some topics, such as supply chain sustainability or renewable energy, correlate with improvements in environmental KPIs, whereas others—such as emissions or electric vehicles—show no significant association. This topic-dependent pattern aligns with emerging calls to move beyond firm-level generalisations about symbolic versus substantive sustainability communication [1,3] and instead consider heterogeneity in environmental issues and implementation pathways. Our findings shift the conceptualisation of the word–action gap toward a more nuanced, content-dependent view.

We identify 34 main topics in ESG reports, grouped into six thematic clusters, with environmental themes accounting for roughly 18.5% of report content. Our results show how topic prevalence has evolved in line with regulatory shifts (e.g., EU climate policy and “double materiality”) and how different sectors emphasise different environmental narratives. Environmental topics, particularly on GHG emissions (T3) and electric vehicles (T14), typically demonstrate an upward trend reflecting the growing stringency of environmental regulation within the EU. More importantly, we show that not all environmental reporting is equal in substance. While some topics (e.g., sustainable value chains and renewable energy) are significantly associated with improvements in environmental KPIs, others (e.g., GHG emissions and electric vehicles) show no such link.

These findings address our central research questions by revealing which topics are emphasised in ESG reports, how they differ across time and sectors, and—critically—how they correlate with measurable environmental outcomes. We thus provide the first large-scale empirical test of the alignment between ESG communication and real-world environmental performance.

Theoretical contributions of our study lie in bridging the gap between ESG discourse and environmental action. We move the literature beyond descriptive mappings of ESG themes toward an empirical evaluation of whether ESG reports reflect substantive or symbolic practices.

This also contributes to the broader literature on greenwashing by showing that the alignment between ESG narratives and measurable environmental outcomes varies systematically across environmental themes. While such heterogeneity is consistent with distinctions between more substantive and more symbolic reporting, it likely reflects a combination of disclosure strategy, sectoral positioning, and implementation dynamics. While some topics show no significant association with environmental KPIs, these findings should not be interpreted as definitive evidence of greenwashing. An absence of correlation can have multiple explanations. Such patterns may also reflect measurement noise in environmental KPIs, sectoral heterogeneity in how environmental initiatives translate into measurable outcomes, or lagged implementation effects where strategic initiatives require several years before affecting observable environmental performance. Many environmental initiatives operate on long time horizons: electrification strategies, value-chain transitions, and emissions reduction plans often require multi-year investments before measurable impacts appear [9]. Firms may also report extensively on issues that are strategically important or stakeholder-salient even when internal implementation is at an early stage [2]. Moreover, environmental KPIs may themselves be imperfect or lagged indicators of underlying environmental action, reflecting measurement constraints documented in recent ESG research [10,11].

Our results therefore indicate that a lack of measured association calls for *cautious interpretation* rather than a direct inference of symbolic disclosure. They highlight the importance of distinguishing between environmental topics that currently align with observable improvements and those for which impacts may exist but are not yet detectable in available data. This resonates with recent work urging caution in equating non-significant findings with greenwashing, particularly in domains where technological, organisational, and regulatory processes unfold over extended timeframes [12,13].

Our study shows that the presence of ESG narratives in corporate reports does not guarantee real progress. Only certain topics are statistically aligned with environmental improvement, suggesting that scrutiny should focus on the type of ESG content communicated—not just its quantity.

Our findings have several implications for policymakers, regulators, and practitioners. First, they suggest that mandatory disclosure frameworks such as the CSRD and SFDR may need to be complemented by requirements that ensure not only standardised reporting structure but also greater transparency about implementation progress. Regulators and standard setters could encourage firms to complement narrative disclosures with topic-specific implementation indicators (e.g., supply chain audits completed, renewable capacity installed, emission reduction measures initiated) that allow stakeholders to evaluate the link between reported commitments and operational outcomes.

Second, investors and ESG analysts may benefit from integrating topic-level patterns into their assessment frameworks. Rather than treating all environmental disclosures as equivalent, analyses could weight topics based on empirically observed associations with environmental outcomes. This aligns with recent recommendations in the sustainable finance literature calling for sharper differentiation between disclosure types when evaluating ESG credibility and investment relevance [5,10].

Third, firms can improve the transparency of their reporting by clarifying time horizons, intermediate milestones, and expected pathways from commitments to environmental impact. For topics where impacts are long term—such as electrification or emissions reduction—firms could complement lagging indicators (e.g., annual emissions data) with *leading indicators* that document operational progress. Recent work emphasises that such indicators can help reduce misinterpretation of ESG reports and provide a clearer basis for evaluating environmental credibility [9,11].

Future research could build on our approach in at least three ways. First, it could conduct a structured comparison between EU firms subject to CSRD and US firms potentially subject to the SEC’s proposed climate disclosure rules, providing a natural institutional contrast in reporting depth and verification requirements. Comparative extensions to Asian markets—especially Japan’s mandatory sustainability disclosure reforms or Singapore’s SGX climate-reporting guidelines—would further illuminate how different regulatory architectures shape the relationship between ESG communication and environmental performance. Additionally, one could examine countries with weaker or voluntary disclosure systems, such as India, Brazil, or South Africa, to assess whether topic–KPI alignment differs in institutional environments with lower levels of standardisation and enforcement. Longitudinal research could also evaluate how topic–performance links evolve as CSRD becomes fully implemented, enabling pre/post policy evaluation of whether heightened reporting requirements improve the correlation between ESG narratives and environmental outcomes.

Second, incorporating more granular and operationally specific environmental KPIs—such as such as Scope 3 value-chain emissions, water-use intensity, landfill diversion rates, or circularity indicators—could further refine topic-performance links. These measures map more directly onto particular report topics (e.g., sustainable value chains) and could therefore sharpen the identification of when ESG disclosure reflects substantive environmental action rather than high-level narrative commitments. The method presented in this study is adaptable to the inclusion of those new KPIs, provided that such indicators become systematically available across firms.

Third, qualitative follow-ups could assess the ambition or credibility of companies’ language around sustainability, providing a richer interpretation of the topics identified here.

A key limitation of our study is its correlational nature of its regression analysis: although we document systematic associations between topic prevalence in ESG reports and changes in environmental KPIs, our analysis does not establish causal effects. The links we identify therefore should not be interpreted as evidence that reporting choices cause improvements in environmental performance. Unobserved strategic decisions, investment cycles, regulatory shocks, or reverse causality may partly drive the observed relationships. Future work using longer panel data and quasi-experimental designs, or policy-induced variation (e.g., CSRD implementation) may help establish causal pathways more convincingly. Still, the present study presents an important step in relating what companies communicate in their ESG reports with their actual environmental performance, stimulating further research in this direction.

A limitation related to topic modelling is that TM allows us to quantify how much space ESG reports devote to different environmental issues, but it does not evaluate the quality, specificity, or ambition of this discussion. The underlying assumption is that greater emphasis in reporting signals strategic priority, yet some firms may devote considerable text to broad themes without detailing concrete measures—something that qualitative analyses could complement. Moreover, because our approach relies on the textual content as reported by firms, it is sensitive to differences in disclosure style, terminology, and reporting practices across companies and sectors. KPI data may also vary in completeness or measurement precision, which can introduce noise into the associations we estimate.

In sum, this study connects ESG narratives with environmental outcomes. While ESG reports are a valuable source of transparency, our results call for greater scrutiny of how much these reports reflect actions versus aspirations. By linking textual content to environmental KPIs, we offer a scalable and replicable method to assess the credibility of sustainability communication—and a foundation for more accountable ESG reporting going forward.

## Supporting information

S1 File Appendix A: Additional results. (ZIP)

S2 File Appendix B: Robustness test. (ZIP)

## References

[1] Bromley P, Powell WW. From smoke and mirrors to walking the talk: decoupling in the contemporary world. ANNALS. 2012;6(1):483–530. doi: 10.5465/19416520.2012.684462

[2] Lyon TP, Maxwell JW. Greenwash: corporate environmental disclosure under threat of audit. J Econ Manag Strat. 2011;20(1):3–41. doi: 10.1111/j.1530-9134.2010.00282.x

[3] Delmas MA, Burbano VC. The drivers of greenwashing. California Manag Rev. 2011;54(1):64–87. doi: 10.1525/cmr.2011.54.1.64

[4] Christensen DM, Serafeim G, Sikochi A. Why is corporate virtue in the eye of the beholder? The case of ESG ratings. Account Rev. 2021;97(1):147–75. doi: 10.2308/tar-2019-0506

[5] Berg F, Kölbel JF, Rigobon R. Aggregate confusion: the divergence of ESG ratings. Review of Finance. 2022;26(6):1315–44. doi: 10.1093/rof/rfac033

[6] Yang BM, Yang OS. Assessing the effect of dynamic capabilities on the ESG reporting and corporate performance relationship with topic modeling: evidence from global companies. Front Psychol. 2022;13:898935. doi: 10.3389/fpsyg.2022.898935 35645900 PMC9130930

[7] Wang F, Zhou X, Gan T. Can green funds improve corporate environmental, social, and governance performance? Evidence from Chinese-listed companies. PLoS One. 2024;19(3):e0301395. doi: 10.1371/journal.pone.0301395 38547225 PMC10977774

[8] Dyer T, Lang M, Stice-Lawrence L. The evolution of 10-K textual disclosure: evidence from latent dirichlet allocation. J Account Economics. 2017;64(2–3):221–45. doi: 10.1016/j.jacceco.2017.07.002

[9] Dimitriadis KA, Koursaros D, Savva CS. The influence of the ‛environmentally-friendly’ character through asymmetries on market crash price of risk in major stock sectors. SSRN Journal. 2025. doi: 10.2139/ssrn.5117489

[10] Serafeim G. ESG: from process to product. 2023.

[11] OECD. Behind ESG ratings: unpacking sustainability metrics. Paris: OECD Publishing; 2025. doi: 10.1787/3f055f0c-en

[12] Martiny A, Taglialatela J, Testa F, Iraldo F. Determinants of environmental social and governance (ESG) performance: a systematic literature review. J Clean Prod. 2024;456:142213. doi: 10.1016/j.jclepro.2024.142213

[13] Sneideriene A, Legenzova R. Greenwashing prevention in environmental, social, and governance (ESG) disclosures: a bibliometric analysis. Research in International Business and Finance. 2025;74:102720. doi: 10.1016/j.ribaf.2024.102720

[14] Hao X, Tian T, Dong L, Wong CWY, Lai K. Unmasking greenwashing in ESG disclosure: insights from evolutionary game analysis. Ann Oper Res. 2025. doi: 10.1007/s10479-025-06538-3

[15] Boiral O. Accounting for the unaccountable: biodiversity reporting and impression management. J Bus Ethics. 2016;135(4). doi: 10.1007/s10551-014-2497-9

[16] Zu Ermgassen SOSE, Howard M, Bennun L, Addison PFE, Bull JW, Loveridge R, et al. Are corporate biodiversity commitments consistent with delivering ‘nature-positive’ outcomes? A review of ‘nature-positive’ definitions, company progress and challenges. J Cleaner Prod. 2022;379:134798. doi: 10.1016/j.jclepro.2022.134798

[17] Michelon G, Pilonato S, Ricceri F. CSR reporting practices and the quality of disclosure: an empirical analysis. Critical Persp Account. 2015;33:59–78. doi: 10.1016/j.cpa.2014.10.003

[18] Cho CH, Guidry RP, Hageman AM, Patten DM. Do actions speak louder than words? An empirical investigation of corporate environmental reputation. Account Organizat Soc. 2012;37(1):14–25. doi: 10.1016/j.aos.2011.12.001

[19] Corporate sustainability reporting. Accessed 2025 November 26. https://finance.ec.europa.eu/capital-markets-union-and-financial-markets/company-reporting-and-auditing/company-reporting/corporate-sustainability-reporting_en

[20] European Commission. Environmental, social and governance (ESG) ratings: Council greenlights new regulation. Accessed 2025 November 26. https://www.consilium.europa.eu/en/press/press-releases/2024/11/19/environmental-social-and-governance-esg-ratings-council-greenlights-new-regulation/

[21] European Parliament. SFDR - Regulation (EU) 2019/2088 of the European Parliament and of the Council on sustainability‐related disclosures in the financial services sector. OJ L. 2019. http://data.europa.eu/eli/reg/2019/2088/oj

[22] The enhancement and standardization of climate-related disclosures for investors. Accessed 2025 December 2. https://www.sec.gov/rules-regulations/2024/03/s7-10-22

[23] IFRS - IFRS foundation announces international sustainability standards board, consolidation with CDSB and VRF, and publication of prototype disclosure requirements. Accessed 2025 December 2. https://www.ifrs.org/news-and-events/news/2021/11/ifrs-foundation-announces-issb-consolidation-with-cdsb-vrf-publication-of-prototypes/

[24] Krueger P, Sautner Z, Tang DY, Zhong R. The effects of mandatory ESG disclosure around the world. J Account Res. 2024;62(5):1795–847. doi: 10.1111/1475-679x.12548

[25] Auzepy A, Tönjes E, Lenz D, Funk C. Evaluating TCFD reporting-A new application of zero-shot analysis to climate-related financial disclosures. PLoS One. 2023;18(11):e0288052. doi: 10.1371/journal.pone.0288052 37917605 PMC10621856

[26] Task Force on Climate-Related Financial Disclosures. Recommendations. Accessed 2025 December 2. https://www.fsb-tcfd.org/recommendations/

[27] European Parliament. CSRD. https://www.europarl.europa.eu

[28] Goloshchapova I, Poon S-H, Pritchard M, Reed P. Corporate social responsibility reports: topic analysis and big data approach. Euro J Finance. 2019;25(17):1637–54. doi: 10.1080/1351847x.2019.1572637

[29] Székely N, Vom Brocke J. What can we learn from corporate sustainability reporting? Deriving propositions for research and practice from over 9,500 corporate sustainability reports published between 1999 and 2015 using topic modelling technique. PLoS One. 2017;12(4):e0174807. doi: 10.1371/journal.pone.0174807 28403158 PMC5389611

[30] Schimanski T, Reding A, Reding N, Bingler J, Kraus M, Leippold M. Bridging the gap in ESG measurement: using NLP to quantify environmental, social, and governance communication. Finance Res Letters. 2024;61:104979. doi: 10.1016/j.frl.2024.104979

[31] Funk C, Tönjes E, Haas C. Exploring the predictive capacity of ESG sentiment on official ratings: a few-shot learning perspective. MAGKS joint discussion paper series in economics. 2024. https://www.econstor.eu/handle/10419/301239

[32] Ul‐Durar S, Dimitriadis KA, Arshed N, De Sisto M, Harati H. Distributional and tail‐dependent perspectives in economic relationships: a review of quantile regression application. J Econ Surveys. 2025. doi: 10.1111/joes.70057

[33] Baier P, Berninger M, Kiesel F. Environmental, social and governance reporting in annual reports: a textual analysis. Financial Market. 2020;29(3):93–118. doi: 10.1111/fmii.12132

[34] Bowen S, Min Y. Analyzing ESG news articles and research papers through LDA (Latent Dirichlet Allocation). APJCRI. 2023;9(8):73–85. doi: 10.47116/apjcri.2023.08.07

[35] Risi D, Schlindwein E. Corporate social responsibility und ESG kombinieren. Zeitschrift für Organisationsforschung. 2024;(06/2024):357–62.

[36] Savin I, van den Bergh J. Opinions of EU citizens about climate policy in their own words. Ecological Economics. 2026;244:108968. doi: 10.1016/j.ecolecon.2026.108968

[37] De Vries E, Schoonvelde M, Schumacher G. No longer lost in translation: evidence that Google Translate works for comparative bag-of-words text applications. Polit Anal. 2018;26(4):417–30. doi: 10.1017/pan.2018.26

[38] Maier D, Baden C, Stoltenberg D, De Vries-Kedem M, Waldherr A. Machine translation Vs. multilingual dictionaries assessing two strategies for the topic modeling of multilingual text collections. Commun Methods Measures. 2021;16(1):19–38. doi: 10.1080/19312458.2021.1955845

[39] Sebo P, de Lucia S. Performance of machine translators in translating French medical research abstracts to English: a comparative study of DeepL, Google Translate, and CUBBITT. PLoS One. 2024;19(2):e0297183. doi: 10.1371/journal.pone.0297183 38300946 PMC10833527

[40] Blei DM. Probabilistic topic models. Commun ACM. 2012;55(4):77–84. doi: 10.1145/2133806.2133826

[41] Savin I, van den Bergh J. Main topics in EIST during its first decade: a computational-linguistic analysis. Environ Innov Soc Transit. 2021;41:10–7. doi: 10.1016/j.eist.2021.06.006

[42] Savin I. Evolution and recombination of topics in technological forecasting and social change. Technol Forecast Social Change. 2023;194:122723. doi: 10.1016/j.techfore.2023.122723

[43] Savin I, Teplyakov N. Topics of the nationwide phone-ins with Vladimir Putin and their role for public support and Russian economy. Inform Process Manag. 2022;59(5):103043. doi: 10.1016/j.ipm.2022.103043

[44] Huang AH, Lehavy R, Zang AY, Zheng R. Analyst information discovery and interpretation roles: a topic modeling approach. Manag Sci. 2018;64(6):2833–55. doi: 10.1287/mnsc.2017.2751

[45] Roberts ME, Stewart BM, Tingley D, Lucas C, Leder‐Luis J, Gadarian SK, et al. Structural topic models for open‐ended survey responses. Am J Political Sci. 2014;58(4):1064–82. doi: 10.1111/ajps.12103

[46] Roberts ME, Stewart BM, Tingley D. stm: an R package for structural topic models. J Stat Soft. 2019;91(2). doi: 10.18637/jss.v091.i02

[47] Boyd-Graber J, Hu Y, Mimno D. Applications of topic models. INR. 2017;11(2–3):143–296. doi: 10.1561/1500000030

[48] Savin I, King LC, Van Den Bergh J. Analysing content of Paris climate pledges with computational linguistics. Nat Sustain. 2025;8(3):297–306. doi: 10.1038/s41893-024-01504-6

[49] Devlin J, Chang MW, Lee K, Toutanova K. BERT: pre-training of deep bidirectional transformers for language understanding. arXiv. 2018. doi: 10.48550/ARXIV.1810.04805

[50] Savin I, Drews S, Maestre-Andrés S, van den Bergh J. Public views on carbon taxation and its fairness: a computational-linguistics analysis. Climatic Change. 2020;162(4):2107–38. doi: 10.1007/s10584-020-02842-y

[51] Savin I, Chukavina K, Pushkarev A. Topic-based classification and identification of global trends for startup companies. Small Bus Econ (Dordr). 2023;60(2):659–89. doi: 10.1007/s11187-022-00609-6 38624813 PMC8886868

[52] Tvinnereim E, Fløttum K. Explaining topic prevalence in answers to open-ended survey questions about climate change. Nature Clim Change. 2015 Aug;5(8):744–7. doi: 10.1038/nclimate2663

[53] Boyd-Graber J, Hu Y, Mimno D. Applications of topic models. Inform Retrieval. 2017;11(2–3):143–296.

[54] Driscoll JC, Kraay AC. Consistent covariance matrix estimation with spatially dependent panel data. Revf Econ Stat. 1998;80(4):549–60. doi: 10.1162/003465398557825

[55] Dosi G, Moschella D, Pugliese E, Tamagni F. Productivity, market selection, and corporate growth: comparative evidence across US and Europe. Small Bus Econ. 2015;45(3):643–72. doi: 10.1007/s11187-015-9655-z

[56] Sarkar S, Searcy C. Zeitgeist or chameleon? A quantitative analysis of CSR definitions. J Cleaner Prod. 2016;135:1423–35. doi: 10.1016/j.jclepro.2016.06.157

[57] Adams C, Alhamood A, He X, Tian J, Wang L, Wang Y. The double-materiality concept: application and issues. Global Reporting Initiative; 2021.

[58] Beede DN, Julian TA, Langdon D, McKittrick G, Khan B, Doms ME. Women in STEM: A gender gap to innovation. SSRN Journal. 2011. doi: 10.2139/ssrn.1964782

[59] Kahn S, Ginther D. Women and STEM. Cambridge, MA: National Bureau of Economic Research; 2017. doi: 10.3386/w23525

[60] Herrmann JK, Savin I. Optimal policy identification: insights from the German electricity market. Technol Forecast Soc Change. 2017;122:71–90. doi: 10.1016/j.techfore.2017.04.014

[61] Hailemariam A, Ivanovski K, Dzhumashev R. Does R&D investment in renewable energy technologies reduce greenhouse gas emissions?. Appl Energy. 2022;327:120056. doi: 10.1016/j.apenergy.2022.120056

