# Supplementary material for: From words to action? Linking ESG reports to environmental performance
Source: PLoS One. 2026 Jun 16;21(6):e0350762. doi: 10.1371/journal.pone.0350762 (PMC13271466; doi:10.1371/journal.pone.0350762)
Supplement: S1 File — (ZIP) [file pone.0350762.s001.zip › Appendix A.docx]

# Appendix A. Additional results

| Country | 2010 | 2011 | 2012 | 2013 | 2014 | 2015 | 2016 | 2017 | 2018 | 2019 | 2020 | 2021 | 2022 | 2023 |
| --- | --- | --- | --- | --- | --- | --- | --- | --- | --- | --- | --- | --- | --- | --- |
| Austria | 7 | 7 | 7 | 7 | 7 | 7 | 8 | 8 | 8 | 8 | 8 | 5 | 8 | 7 |
| Belgium | 10 | 10 | 11 | 11 | 12 | 12 | 16 | 14 | 15 | 16 | 16 | 7 | 17 | 16 |
| Denmark | 13 | 14 | 14 | 14 | 14 | 17 | 19 | 19 | 20 | 20 | 22 | 21 | 28 | 28 |
| Finland | 10 | 9 | 10 | 11 | 11 | 12 | 14 | 15 | 15 | 15 | 16 | 7 | 15 | 15 |
| France | 45 | 48 | 53 | 52 | 56 | 59 | 68 | 68 | 70 | 73 | 74 | 62 | 80 | 71 |
| Germany | 45 | 44 | 44 | 43 | 48 | 49 | 63 | 65 | 68 | 68 | 88 | 166 | 180 | 140 |
| Ireland | 5 | 5 | 5 | 4 | 5 | 6 | 7 | 7 | 7 | 7 | 7 | 5 | 8 | 9 |
| Italy | 14 | 18 | 20 | 22 | 22 | 23 | 27 | 27 | 27 | 27 | 30 | 25 | 32 | 29 |
| Netherlands | 14 | 16 | 17 | 18 | 23 | 22 | 27 | 26 | 28 | 30 | 33 | 26 | 34 | 32 |
| Norway | 12 | 13 | 13 | 15 | 15 | 15 | 16 | 16 | 16 | 17 | 18 | 11 | 19 | 17 |
| Poland | 1 | 2 | 2 | 3 | 4 | 4 | 6 | 6 | 6 | 6 | 8 | 6 | 10 | 9 |
| Portugal | 2 | 2 | 3 | 3 | 3 | 3 | 4 | 4 | 4 | 4 | 4 | 1 | 4 | 4 |
| Spain | 13 | 17 | 18 | 21 | 22 | 22 | 25 | 25 | 25 | 25 | 27 | 24 | 28 | 26 |
| Sweden | 45 | 44 | 47 | 50 | 52 | 55 | 58 | 62 | 62 | 62 | 64 | 51 | 71 | 63 |
| Switzerland | 33 | 35 | 36 | 37 | 39 | 40 | 49 | 50 | 50 | 52 | 53 | 31 | 55 | 53 |
| United Kingdom | 98 | 101 | 100 | 103 | 106 | 114 | 127 | 128 | 134 | 136 | 149 | 84 | 157 | 139 |

Table A1. ESG reporting Navigator Document Database Summary

Table A2. Data description and sources

|  | Description | Units | Source |
| --- | --- | --- | --- |
| Year | Year when the report was published. | - | ESG reporting Navigator |
| Country | Country where the company that released the report is based. | - |  |
| Sector | The main sector in which each company operates. | - |  |
| Sustainability report | Report highlighting companies’ efforts in terms of ESG performance | - |  |
| CO2 Emissions | Total carbon dioxide (CO2) and CO2 equivalents emission in tonnes. | Tonnes | Refinitiv Workspace |
| Emission intensity (CO2 emissions to revenues) | Total CO2 and CO2 equivalents emission in tonnes divided by revenue in US dollars in million. | Tonnes/ revenues in USD million |  |
| Energy Total | Has the company set targets or objectives to be achieved on emission reduction? | Gigajoules |  |
| Energy intensity  (Energy to revenues) | Energy use divided by company revenues. | Gigajoules/ revenues in USD million |  |
| ESG score | Overall company score based on the self-reported information in the environmental, social and corporate governance pillars. | 0 to 100 |  |
| Emissions score | Emission category score measures a company's commitment and effectiveness towards reducing environmental emission in the production and operational processes. | 0 to 100 |  |
| Environmental innovation score | Environmental innovation category score reflects a company's capacity to reduce the environmental costs and burdens for its customers, and thereby creating new market opportunities through new environmental technologies and processes or eco-designed products. | 0 to 100 |  |
| Revenues | Total revenues for the company in that specific year | USD million |  |

Table A3. Number of ESG reports by sector.

| Sector | Number of reports | % of reports |
| --- | --- | --- |
| Automobiles & Other Transport Vehicles | 33 | 2.23% |
| Basic Materials & Mining | 60 | 4.06% |
| Biotechnology & Pharmaceuticals | 74 | 5.01% |
| Building & Construction | 92 | 6.23% |
| Chemicals, Fuels & Biofuels | 38 | 2.57% |
| Clothing & Footwear | 54 | 3.66% |
| Electronics | 40 | 2.71% |
| Energy Production & Gas | 96 | 6.50% |
| Financial Services | 265 | 17.94% |
| Food & Beverages | 95 | 6.43% |
| Forestry & Agriculture | 11 | 0.74% |
| Heavy Machinery | 25 | 1.69% |
| Industrial Machinery | 18 | 1.22% |
| Industrial Products & Services | 89 | 6.03% |
| Insurance | 54 | 3.66% |
| IT & Telecommunication Services | 80 | 5.42% |
| Media & Entertainment | 34 | 2.30% |
| Medical Equipment & Technology | 7 | 0.47% |
| Metals & Glass | 19 | 1.29% |
| Personal Products & Health Care | 36 | 2.44% |
| Real Estate & Hotels | 10 | 0.68% |
| Retail | 48 | 3.25% |
| Software & IT Services | 31 | 2.10% |
| Transportation & Logistics | 40 | 2.71% |
| Water, Electricity & Heating | 128 | 8.67% |

Fig A1. STM models results for different number of topics

Fig A2. Topic prevalence over time.

Fig A3. Dendrogram of six clusters of companies based on topic prevalences related to environment.

Fig A4. Company clusters coloured by country.

Note: Wordclouds showing the different company clusters depending on the topics they address. Font size in a cloud reflects the revenue of the company in 2023, while colour indicates the country: Belgium - France - United Kingdom - Switzerland - Germany - Italy - Spain - Netherlands - Portugal - Norway - Sweden - Denmark - Austria - Ireland - Finland – Poland.
