# Supplementary material for: From words to action? Linking ESG reports to environmental performance
Source: PLoS One. 2026 Jun 16;21(6):e0350762. doi: 10.1371/journal.pone.0350762 (PMC13271466; doi:10.1371/journal.pone.0350762)
Supplement: S2 File — (ZIP) [file pone.0350762.s002.zip › Appendix B.docx]

# Appendix B. Robustness test

To provide a robustness test regarding the choice of the 10000-word threshold used to segment the ESG reports, we present an alternative analysis using a smaller threshold of 3000 words. A lower threshold was selected as the motivation for segmenting texts was to increase the number of observations used to measure co-occurrence between words. At least in theory, a larger number of observations can provide more precise insights. Applying this new threshold, we obtained 13,896 individual documents, which contained 18,944 unique terms and 10,821,824 total word occurrences. As shown in Figure B1, the 34 topics identified in the baseline analysis (reported in the main text) remain consistent. Moreover, all three selection criteria – prediction accuracy, exclusivity and coherence – demonstrate similar performance. This suggests that splitting ESGs into more segments does not improve the quality of the resulting topics in terms of their out-of-sample prediction performance, exclusivity of the key terms in the topics and topic coherence.

Figure B1. Criteria for selecting the number of topics after segmenting ESGs into 13896 texts using the 3000-word threshold.

Figure B1 shows the word clouds for the resulting 34 topics. The same thematic groups emerge with fairly consistent prevalences (see Table B1). Overall, the results of the robustness test are highly consistent with the baseline analysis, indicating that the identified thematic structure is stable and robust to alternative model specifications. Some variation in FREX words across topics is expected due to the stochastic nature of topic modelling and does not affect the underlying thematic interpretation.

Figure B2. Word clouds of 34 topics generated based on ESG texts in the robustness test. Font size in a cloud reflects the probability of the appearance of the respective word given the topic, while a darker shade of colour indicates higher exclusivity of a word

Table B1. Robustness test comparison of thematic groups and constituent topics

| **Original analysis with 10000 words threshold** | | **Robustness test with 3000 words threshold** | |
| --- | --- | --- | --- |
| **Thematic group** | **Constituent topics** | **Thematic group** | **Constituent topics** |
| G1: Organisation governance, 33.22% | T1: Inclusive leadership, 8.47% | G1: Organisation governance, 33.71% | T1: Inclusive leadership, 8.16% |
|  | T2: Supervisory board, 6.73% |  | T2: Supervisory board, 6.72% |
|  | T4: Corporate governance, 5.66% |  | T4: Corporate governance, 5.60% |
|  | T6: Corporate disclosure, 5.65% |  | T6: Corporate disclosure, 6.50% |
|  | T15: Firm growth, 2.81% |  | T15: Firm growth, 3.20% |
|  | T16: Executive compensation, 2.68% |  | T16: Executive compensation, 2.21% |
|  | T25: Shareholder rights, 1.22% |  | T25: Shareholder rights, 1.32% |
| G2: Environment, 18.48% | T3: GHG emissions, 6.62% | G2: Environment, 18.42% | T3: GHG emissions, 6.52% |
|  | T8: Sustainable VCs, 4.90% |  | T8: Sustainable VCs, 4.95% |
|  | T14: Electric vehicles, 3.19% |  | T14: Electric vehicles, 3.23% |
|  | T20: Renewable energy, 1.81% |  | T20: Renewable energy, 1.77% |
|  | T27: Energy sources, 1.00% |  | T27: Energy sources, 1.02% |
|  | T28: Subsidies & environment, 0.96% |  | T28: Subsidies & environment, 0.93% |
| G3: Sector-specific issues, 15.78% | T10: Affordable housing, 4.24% | G3: Sector-specific issues, 15.72% | T10: Affordable housing, 4.04% |
|  | T12: Consumables, 3.72% |  | T12: Consumables, 3.82% |
|  | T17: Natural resources, 2.28% |  | T17: Natural resources, 2.31% |
|  | T22: Healthcare, 1.49% |  | T22: Healthcare, 1.49% |
|  | T23: Real estate, 1.42% |  | T23: Real estate, 1.45% |
|  | T26: Public utilities, 1.00% |  | T26: Public utilities, 1.10% |
|  | T29: Telecommunication, 0.85% |  | T29: Telecommunication, 0.75% |
|  | T30: Aquaculture, 0.78% |  | T30: Aquaculture, 0.76% |
| G4: People and community, 13.07% | T5: Young customers, 5.65% | G4: People and community, 13.16% | T5: Young customers, 5.61% |
|  | T9: Women rights, 4.70% |  | T9: Women rights, 4.60% |
|  | T18: Cultural identity, 2.09% |  | T18: Cultural identity, 2.29% |
|  | T31: Retail, 0.64% |  | T31: Retail, 0.66% |
| G5: Financial aspects, 11.61% | T11: Risk management, 4.14% | G5: Financial aspects, 11.40% | T11: Risk management, 4.11% |
|  | T13: Asset value, 3.62% |  | T13: Asset value, 3.51% |
|  | T19: Profits & taxation, 1.97% |  | T19: Profits & taxation, 1.77% |
|  | T24: Financial disclosure, 1.25% |  | T24: Financial disclosure, 1.34% |
|  | T33: Financial risks, 0.39% |  | T33: Financial risks, 0.41% |
|  | T34: Insurance, 0.24% |  | T34: Insurance, 0.26% |
| G6: Public regulation, 7.84% | T7: Ethics and compliance, 5.48% | G6: Public regulation, 7.59% | T7: Ethics and compliance, 5.33% |
|  | T21: Accreditation, 1.77% |  | T21: Accreditation, 1.72% |
|  | T32: Legal proceedings, 0.59% |  | T32: Legal proceedings, 0.54% |
