# Supplementary material for: From words to action? Linking ESG reports to environmental performance
Source: PLoS One. 2026 Jun 16;21(6):e0350762. doi: 10.1371/journal.pone.0350762 (PMC13271466; doi:10.1371/journal.pone.0350762)
Supplement: Attachment — Submitted filename: Review on manuscript ESG Reports .pdf [file pone.0350762.s005.pdf]

Reviewer Report
Manuscript Title: *From Words to Action? Linking ESG Reports to Environmental Performance*
Journal: *PLOS One*
Manuscript Number: *PONE-D-25-44096R1*

General Information:

The article examines whether what firms say in their ESG reports about environmental priorities translates into measurable environmental improvements. Using computational linguistics (structural topic modelling) on 1,477 ESG reports from STOXX Europe 600 firms (2010-2023), the authors identify key environmental topics and then test whether emphasizing (or increasing emphasis on) specific topics is associated with better environmental performance indicators (e.g., CO_2 / emissions intensity, energy intensity, and Refinitiv environmental scores). They find the 'talk-action' link is topic-dependent: themes like sustainable value chains and renewable energy are more aligned with improved environmental KPIs, while heavy discussion of topics like emissions or electric vehicles often shows no significant performance association.

Major Comments:

I suggest the authors answer the following suggestions based on my guidelines to improve the research article and for publication, which are the following:

1. How sensitive are the 34 STM topics (especially the 6 environmental ones) to the preprocessing choices (lemmatization, bigrams, stop-words) and the selected topic number criteria (held-out likelihood, exclusivity, semantic coherence)?

2. The literature review should be presented though in a clearer manner how the present study differentiates from existing research and makes a step towards better understanding the research problem. I recommend the authors to consult the following survey and empirical papers to contextualize your findings. This should help the readers to understand the novelty of your work (use the following references which are updated(recent) in the literature review section):

    - Dimitriadis, K. A., Koursaros, D., & Savva, C. S. (2024). The influence of the "environmental-friendly" character through asymmetries on market crash price of risk in major stock sectors. *Journal of Climate Finance*, *9*, 100052.

    - Ul-Durar, S., Dimitriadis, K. A., Arshed, N., De Sisto, M., & Harati, H. (2025). Distributional and Tail-Dependent Perspectives in Economic Relationships: A Review of Quantile Regression Application. *Journal of Economic Surveys*.

3. Also, the paper needs to improve the quality of the results section and how to present things (to be more explicit in discussing results, not so extensive, use the following article in the references section based on this:
    - https://www.sciencedirect.com/science/article/abs/pii/S0264999325001907)

4. The authors should give more emphasis on the importance of their and should also be clearer in the conclusion section. Overall, this paper is interesting and well-structured, and it is my belief that it will be appealing for the readers of the journal. Furthermore, the abstract it would be better to be one whole paragraph which includes the most important result, the innovation of the research article and what does this article study.

5. The paper should clearly address the following: What is the research question? Why is this question important and interesting? How did they approach the problem?

6. The reports are split into ≤ 10,000-word segments and then re-aggregated0-how do results change under alternative thresholds (e.g., 3,000, full-document) and are the aggregation weights fully justified?

7. None-English ESG reports are translated with DeepL-did the authors test whether translation affects topic prevalence (e.g., by re-running STM on an English-only subsample)?

8. Environmental KPIs from Refinitiv are only available for 2019-2023-does this short window provide enough within-firm variation to support the 'words-to-action' claims?

9. Given the regressions link topic prevalence/changes to changes in KPIs with firm and year fixed effects, what remaining endogeneity risks exist (reverse causality, omitted time-varying factors), and how are they mitigated?

10. For topics like 'GHG emissions' or 'electric vehicles' with insignificant links to KPIs, how do the authors distinguish 'symbolic disclosure/greenwashing' from alternative explanations (measurement noise, sector heterogeneity, lagged effects)?

Recommendation:

Finally, I think this article with these comments will get better and will be published. Therefore, this manuscript should undergo minor revisions.
