# Supplementary material for: From words to action? Linking ESG reports to environmental performance
Source: PLoS One. 2026 Jun 16;21(6):e0350762. doi: 10.1371/journal.pone.0350762 (PMC13271466; doi:10.1371/journal.pone.0350762)
Supplement: Attachment — Submitted filename: Review 2 on manuscript Words to Action.pdf [file pone.0350762.s008.pdf]

**Reviewer Report**
**Manuscript Title**: *From Words to Action? Linking ESG Reports to Environmental Performance*
**Journal:** *PLOS One*
**Manuscript Number**: *PONE-D-25-44096R2*

## General Information:

The new version of this paper is well-improved. The authors properly revised the paper by adding more convincing evidence. Here are some minor suggestions based on the latest version of the paper, which are listed as follows in order of appearance.

## Minor Comments:

1. The literature review should be presented though in a clearer manner how the present study differentiates from existing research and makes a step towards better understanding the research problem. I recommend the authors to consult the following survey and empirical papers to contextualize your findings. This should help the readers to understand the novelty of your work (literature review section):

    - Kyriazis, N. A., Dimitriadis, K. A., & Theodossiou, P. (2026). Can Banking Intermediates Crowd Out Their High-Tech Promising Successors? A Financial Stress Perspective. *International Journal of Finance & Economics*.

2. I want to see the innovation in this study from the previous studies, to see the difference between them. The research gap that is covered in this research article. Please incorporate these to be clear in the abstract, introduction and the conclusion of the paper.

3. How robust is the causal interpretation of the relationship between ESG topic emphasis and environmental KPIs, given that the analysis is based on contemporaneous associations rather than long-term dynamics?

4. To what extent does the use of structural topic modelling (STM) accurately capture meaningful environmental themes, and how sensitive are the results to the chosen number of topics and preprocessing steps?

5. How do the authors address potential measurement inconsistencies in environmental KPIs (e.g., Refinitiv scores vs firm-reported data) when linking ESG reporting content to actual environmental performance?

## Recommendation:

Finally, I think this article with these comments will get better and will be published. Therefore, this manuscript should undergo minor revisions.
