# Supplementary material for: From words to action? Linking ESG reports to environmental performance
Source: PLoS One. 2026 Jun 16;21(6):e0350762. doi: 10.1371/journal.pone.0350762 (PMC13271466; doi:10.1371/journal.pone.0350762)
Supplement: Attachment — Submitted filename: 251212 Response to Reviewers FINAL.docx [file pone.0350762.s007.docx]

**Response to Reviewers**

Manuscript title: [From Words to Action? Linking Sustainability Reports to Environmental Performance

Manuscript ID: P PONE-D-25-44096 PLOS ONE
Corresponding author: Ivan Savin

We sincerely thank both reviewers for their thoughtful, constructive, and highly valuable feedback. Their comments provided clear guidance on how to sharpen the theoretical framing, strengthen the methodological transparency, and improve the clarity and relevance of the manuscript.

In response, we undertook substantial revisions across the Introduction, Literature Review, Data and Methods, Results, Discussion, and Conclusion sections. These include: a fully reorganized and significantly expanded literature review; a clearer articulation of the research gap and contribution; strengthened methodological explanations, including justifications for key analytic choices; more explicit links between hypotheses and results; a tempered and more nuanced interpretation of findings; and an extended discussion of policy and practical implications as well as study limitations.

We believe these revisions significantly enhance the clarity, rigor, and contribution of the manuscript. Below, we provide a point-by-point response to each comment.

**Response to Reviewer #1**

*(our responses are in italics)*

The manuscript addresses a critically important question in the ESG space with a robust methodological approach but to help strengthen it for publication, I have compiled my main comments below.

*Thank you very much for your positive evaluation and for the constructive and insightful comments. We found your suggestions very helpful, and they have substantially strengthened the manuscript. Below, we address each point in turn.*

**Abstract**

1. Explicitly mention the geographic and regulatory context of the study to immediately frame the generalizability of the findings.

*We have revised the abstract to directly state the European context and relevant regulatory environment, thereby clarifying the scope and generalizability of the findings.*

2. Clarify the nature of the findings by using more precise language and avoid causation.

*We updated the abstract to emphasize associations rather than causal claims, ensuring accurate representation of our empirical analysis.*

**Introduction**

1. Strengthen the opening by defining the research gap more sharply. “Little is known” is a weak choice of words and implies vagueness, articulate it with clarity.

*We refined the introduction to articulate a more specific and evidence-based research gap, replacing vague phrasing with a clear statement of what is missing in the current literature.*

2. Improve the logical flow between the problem of un-audited reports, the practice of greenwashing, and the proposed methodological solution (computational linguistics). The connection should be made more explicit.

*We strengthened the narrative link between the lack of auditing, greenwashing risks, and the computational text analysis approach, clarifying how the method addresses these issues and contributed to faster and more objective analysis of the nexus between ESG reporting and actual actions towards low-carbon transition.*

**Literature Review**

1. Reorganize the literature review into clearly labeled sub-sections to enhance readability and structure.

*We appreciated this suggestion. The literature review has been fully reorganized into four clearly labeled subsections: (2.1) sustainability reporting and variation in environmental communication, (2.2) greenwashing and the persistence of the talk–action gap, (2.3) regulation and rising expectations for meaningful disclosure, and (2.4) analysing ESG reporting content through topic modelling. Each subsection ends with an integrating paragraph highlighting what remains underexplored and how this motivates our study.*

2. Incorporate more recent literature (2021–2025) on greenwashing, the EU's Sustainable Finance Disclosure Regulation (SFDR), and the Corporate Sustainability Reporting Directive (CSRD) to ensure the review reflects the current, rapidly evolving regulatory and academic landscape.

*We have substantially expanded and updated the literature review to reflect recent developments in ESG research and regulation (2021–2025). The revised review now integrates recent empirical and review-based work on ESG reporting heterogeneity, disclosure credibility, and greenwashing (e.g., Christensen et al., 2022; Berg et al., 2022; Martiny et al., 2024; Sneideriene & Legenzova, 2025; Hao et al., 2025), as well as recent methodological and conceptual contributions using textual analysis of ESG disclosures (e.g., Schimanski et al., 2024; Auzepy et al., 2023).*

*In addition, we explicitly incorporate recent regulatory and policy-oriented sources addressing the evolving disclosure landscape, including the EU’s Corporate Sustainability Reporting Directive and Sustainable Finance Disclosure Regulation (European Parliament, 2019; European Commission, 2024), as well as comparative work on mandatory ESG disclosure regimes (Krueger et al., 2024; OECD, 2025).*

**Data and Methods**

1. Provide a more detailed justification for the choice of Structural Topic Modeling (STM) over other methods like LDA. Explicitly state that the ability to incorporate metadata (sector, year) was a key factor, leading to more coherent and context-aware topics.

*We clarified the choice of STM, emphasizing its ability to integrate document-level covariates (e.g., sector, year) for more contextually grounded and coherent topic elicitation and inference.*

2. Offer a clearer rationale for the decision to split reports into 10,000-word chunks. Explain why this approach was necessary for topic quality and how the aggregation back to the report level preserves the integrity of the analysis for the subsequent regression.

*The decision to segment the ESG reports into sections was motivated by a well-known limitation of probabilistic topic models, including STM: topic quality depends on the model’s ability to reliably measure word co-occurrence patterns within documents. When only a small number of very long documents is available, STM has limited data on which to estimate these co-occurrence statistics, often resulting in overly broad or vague topics. This issue is documented and addressed in Savin et al. (2025) in Nature Sustainability, who show that 309 long documents were insufficient for clear topic distinctions and therefore segmented them into 1280 shorter texts to improve semantic coherence and exclusivity of topics.*

*Following this approach, we divided each ESG report into consecutive blocks of up to 10,000 words. This threshold ensured that each segment remained substantively meaningful while increasing the total number of observational units from 1,477 to 4,709. Segmenting long documents is a standard practice in computational linguistics (for example, BERT-based topic models apply a similar procedure, see Devlin, 2019), and substantially improves topic quality because co-occurrence patterns can be estimated more precisely on a larger number of shorter texts. The resulting STM therefore identifies more coherent and interpretable themes than when trained on the original set of very long documents.*

*After estimating STM on the segmented texts, we aggregated topic prevalences back to the original ESG-report level using segment-length weights. This procedure mirrors the aggregation strategy in Savin et al. (2025) and ensures that the derived topic shares accurately reflect the full content of each original report. Because aggregation is performed after STM estimation and preserves each segment’s proportional contribution to the whole document, no information is lost for subsequent econometric analysis. The regression stage therefore uses topic prevalences that are consistent with the original documents, but benefit from the improved topic quality enabled by the segmentation step.*

3. Discuss potential limitations introduced by text pre-processing, such as the loss of nuanced meaning during lemmatization or the removal of tables/figures that might contain crucial environmental data.

*We agree that any text-processing pipeline may remove some nuances. However, our preprocessing steps (lowercasing, stop-word removal, lemmatization, rare-term filtering, and bigram formation) follow standard practice for probabilistic topic models and are well aligned with the assumptions of the Structural Topic Model (STM). STM, like LDA, is a bag-of-words model that relies on word counts and co-occurrences and cannot directly incorporate numeric values, tables, figures, or layout information. Therefore, removing non-textual elements (tables/figures) does not lead to information loss from the model’s perspective, as these elements would not be used by STM.*

*Similarly, lemmatization and stop-word removal are widely recommended to reduce sparsity, merge inflected variants of the same concept, and improve topic coherence without altering the substantive meaning captured by word distributions. Peer-reviewed studies show that such preprocessing improves interpretability and stability of topic models rather than distorting meaning (Boyd-Graber et al., 2017).*

*Overall, while any preprocessing imposes simplification, the steps we used are standard, theoretically appropriate for bag-of-words models, and consistent with best practice in the STM literature (Roberts et al., 2019). We clarified this in the revised manuscript.*

4. In the regression analysis (Equation 2), provide a stronger theoretical and econometric justification for including both the level and the first difference of topic prevalence as independent variables. Acknowledge that this is a non-standard specification and defend its use.

*We agree that including both the level and the first difference of topic prevalence is a non-standard specification, and we now clarify its theoretical and econometric justification in the revised manuscript. Including both the level and the change of topic prevalence follows the logic used in empirical studies such as Dosi et al. (2015), who show that levels and changes of an explanatory variable capture different mechanisms: levels reflect persistent, structural heterogeneity across firms, while changes capture dynamic adjustments that are more directly linked to performance outcomes.*

*In our context, the level of a topic indicates a company’s stable emphasis on an environmental theme, whereas the change reflects year-to-year shifts in disclosure. Omitting either would force us to ignore one of these channels or absorb it into fixed effects. Including both is therefore appropriate, even if non-standard, because it allows us to distinguish persistent disclosure strategies from dynamic adjustments in ESG reporting—exactly the distinction that motivated the dual specification in Dosi et al. (2015). We have added the explanation in the revised manuscript.*

5. Comment on the very low R-squared values in some regressions (e.g., Energy Intensity, ESG Score). Discuss what this implies for the model's explanatory power and the potential for other, unobserved factors driving KPI changes.

*We acknowledge the low R-squared values for some KPI regressions (e.g., energy intensity, ESG score). This is not unexpected given the structure of our empirical analysis. First, KPI changes are driven by many factors that are unrelated to report content and are absorbed neither by topic prevalence nor fixed effects. Consequently, the model is designed to detect whether topic emphasis is systematically associated with KPI changes, not to fully explain overall KPI variation.*

*Second, R-squared values in fixed-effects panel regressions with differenced dependent variables are typically low, especially when KPIs are noisy or updated infrequently—precisely the type of measurement limitations documented in ESG research.*

*Finally, the presence of statistically significant coefficients despite low R-squared values indicates that certain topics still contain meaningful information, even if most KPI variation is driven by unobserved or unmeasured factors. We now clarify this interpretation in the text.*

**Results**

1. Improve the interpretation of statistical results in the text. For each significant coefficient in Table 4, provide a clear, non-technical explanation of what it means (e.g., "A one-percentage-point increase in the prevalence of Topic X is associated with a change of Y in emission intensity").

*We thank the reviewer for this helpful suggestion. We have now expanded the discussion of Table 4 by adding non-technical interpretations of all statistically significant coefficients. In particular, we clarify that the coefficients on topic levels capture how firms with a systematically higher emphasis on a given topic tend to differ in their KPI changes, while the coefficients on topic changes (ΔT) describe how year-to-year shifts in reporting emphasis co-move with KPI changes. For each significant coefficient, we now explicitly state in the text whether it reflects a long-run reporting orientation or a short-run adjustment in topic prevalence and how this relates to the corresponding KPI.*

2. In Table 2, consider adding a column for the number of reports in which each topic is a dominant or significant theme, to give readers a better sense of topic distribution across the corpus.

*Thank you for this valuable comment. We have added a column to Table 3 on the number of reports per topic where this topic is the largest.*

3. Ensure all figures referenced in the main text (Figures 1–7) are included and are of publication quality in the final submission.

*We ensured that all figures are referenced in the main text of the revised manuscripts, are clearly labeled, and formatted to meet publication requirements*

**Discussion**

1. More explicitly state the novel contribution of the study in the opening of the discussion.

*We thank the reviewer for this suggestion. We revised the opening of the Discussion to more explicitly state the study’s conceptual contributions. The revised text now highlights (a) that our analysis identifies topic-level variation in the relationship between ESG disclosures and environmental performance—an aspect not addressed in prior research—and (b) that we reconceptualize the talk–action gap as topic-dependent rather than solely a firm-level characteristic. These changes clarify the novelty of our contribution and establish a stronger link between the empirical findings and the theoretical framing. The revised paragraphs appear at the beginning of Section 5.*

2. Temper the language regarding "greenwashing." The absence of a significant correlation is not definitive proof of greenwashing. Acknowledge alternative explanations, such as reporting on EVs or GHG that have not yet yielded measurable results.

*We agree with the reviewer’s concern and have substantially revised the discussion of non-significant associations. The new text clarifies that an absence of measurable correlation does not constitute evidence of greenwashing. Instead, we acknowledge several alternative explanations, including (a) long time horizons for environmental initiatives such as electrification or emissions reduction; (b) early-stage implementation processes that may not yet influence environmental KPIs; and (c) limitations and lags in environmental performance measurement. These considerations now temper our interpretation and align the findings with recent work warning against equating null results with symbolic disclosure.*

3. Elaborate on the policy and practical implications. Provide specific, actionable recommendations.

*We appreciate this suggestion and have expanded the implications section accordingly. The revised text now provides concrete recommendations for policymakers (e.g., complementing CSRD/SFDR reporting requirements with topic-specific implementation indicators), investors (e.g., incorporating topic-level patterns into ESG evaluation frameworks), and firms (e.g., reporting leading indicators that precede measurable KPI changes). The expanded paragraphs appear in Section 5 toward the end of the Discussion.*

Conclusion

1. Provide more specific and concrete suggestions for future research. Instead of "extending the analysis to other regions," suggest specific regions for comparison (e.g., "a comparative analysis between EU firms under CSRD and US firms under SEC proposed rules").

*Thank you for this comment. We now include several concrete and actionable directions for future research, specifying particular regions, regulatory frameworks (e.g., EU CSRD, US SEC climate disclosure rules), and comparative designs that extend beyond the general suggestion previously provided.*

2. Recommend future studies to use more granular KPIs, such as Scope 3 emissions or water usage intensity, to create even stronger links with specific report topics.

*Thank you for this valuable suggestion. We now explicitly recommend that future work employ more granular environmental performance indicators—such as Scope 3 emissions, water-use intensity, or circularity metrics—to strengthen topic-level links between ESG communication and underlying operational outcomes.*

3. Explicitly state the primary limitation of the study: the correlational nature of the analysis and the inability to make definitive causal claims about the relationship between reporting and performance.

*We appreciate this comment. We now explicitly acknowledge in the conclusion that our empirical design is correlational and does not allow for causal claims about the relationship between ESG reporting and environmental performance. We also clarify the implications of this limitation for interpretation of our findings.*

**Grammar and Writing Style**

1. Maintain the coherence and flow throughout the manuscript. Simplify complex sentence structures, particularly in the Methods and Results sections. For example, break down long sentences explaining the STM process or regression outputs into shorter, more digestible statements.

*We reviewed and revised complex sentences, especially in the Methods and Results sections, to enhance readability while retaining accuracy.*

2. Ensure consistent use of terminology (e.g., stick with "ESG reports" or "sustainability reports" rather than using them interchangeably without need).

*We standardized terminology – ESG reports – throughout the manuscript to ensure consistency.*

3. Ensure the numbering (numeric values format) is consistent in manuscript.

*We harmonized numeric formatting and ensure consistency across all tables, figures, and text in the revised manuscript.*

**Response to Reviewer #2**

*(our responses are in italics)*

*We thank Reviewer 2 for the thoughtful and constructive comments. We found these suggestions very helpful, particularly regarding clarity of hypothesis testing, limitations, and consistency. Below, we respond to each point.*

1. In the end of section 3.2 the authors present two hypotheses (which should also include a null hypothesis?). A clear statement addressing whether to support or refute these two hypotheses should be more clearly made in the Results section. Did the authors use the methods to actually find answers to these particular hypotheses?

*We thank the referee for this suggestion. Our hypotheses indeed imply corresponding null hypotheses (no association between topic prevalence and environmental KPIs), and the statistical tests in Section 4.5 are explicitly evaluating them. In the revised manuscript we (i) explicitly state the null hypotheses at the end of Section 3.2 clarifying that the regression analysis described in equation (2) is meant to test them; (ii) add a short concluding paragraph in Section 4.5 summarizing whether the evidence supports or rejects H1 and H2.*

*.*2. The Conclusions section was missing reflection on the Limitations of this study, which should be included in the manuscript. For instance, is there something that cannot be found out with the used methods, or are there risks of errors, biases etc., and where? Also, how adaptive is this method for when new KPIs are included or if environmental actions are not reported but still impact performance?

*We thank the referee for this helpful comment. We expanded the discussion of limitations discussing among others (i) the correlational nature of our regression analysis, (ii) what topic modelling can and cannot detect in ESG reports and (iii) the adaptability of the method when additional KPIs or reporting standards become available.*

3. I noted a few minor typing mistakes in the manuscript. For example, in Figure 7 the first cluster is titled as Cluster 1 but rest of the clusters are titled as Groups.

*Thank you for spotting this inconsistency. We corrected this and ensured uniform labeling across figures and text.*
