# Supplementary material for: From words to action? Linking ESG reports to environmental performance
Source: PLoS One. 2026 Jun 16;21(6):e0350762. doi: 10.1371/journal.pone.0350762 (PMC13271466; doi:10.1371/journal.pone.0350762)
Supplement: Attachment — Submitted filename: PONE-D-25-44096_R1 Overall Recommendation.docx [file pone.0350762.s004.docx]

**Dear Editor
PLOS ONE**

Thank **you for providing me with the opportunity to review this manuscript** (PONE-D-25-44096_R1)**.**

**Overall Recommendation:** Minor Revision

This is a strong, well-executed study that makes a meaningful contribution to the ESG reporting literature. The revisions have significantly improved the manuscript. I recommend acceptance after minor revisions, focusing on the clarifications and editorial corrections noted below.

After examining the manuscript revision and the prior reviewer’s feedback, I have prepared a structured review in two parts:

**Part 1** – Assessment of Authors’ Response to Previous Reviewer Comments
Overall, the authors have made substantial and thoughtful revisions to the manuscript. They have:

a. Reorganized and expanded the literature review significantly.
b. Clarified the research gap and contributions.
c. Strengthened methodological transparency, including justifications for analytical choices.
d. Improved the linkage between hypotheses and results.
e. Provided a more nuanced discussion of findings, limitations, and implications.

The manuscript is now more rigorous, coherent, and impactful. However, some areas still require minor clarification or correction.

**Part 2** – Independent Comments

**Title and Abstract**

1. Consider slightly rephrasing the abstract to emphasize the *methodological novelty* (topic modeling + KPI linkage) upfront.

**Data and Methods**

2. The handling of long reports by splitting into 10,000-word segments is reasonable but should include a brief robustness note in the main text (not just the appendix).
3. The regression specification (Equation 2) is sound, but please clarify why both level and change in topic prevalence are included in the same model—consider a brief note on multicollinearity checks.

**Results**
4. In Table 4, some coefficient signs are counterintuitive (e.g., T8 Sustainable VCs positive for emission intensity). Please provide a brief interpretation in text.
5. The discussion of why certain topics (e.g., GHG emissions, electric vehicles) show no significant KPI association is appropriately cautious and nuanced.

**References and Supplementary Material**

6. Appendix tables and figures are helpful; ensure all are referenced in the main text.

**Minor Editorial Issues**
7. Some formatting inconsistencies remain (e.g., “organisation” vs. “organization,” figure callouts like “Figure 4.4.”). Please standardize.
8. A few typos/grammatical errors persist (e.g., p. 84 “sizesise” → “size”). A final proofread is recommended.

9. All text should be in the same font style (headings are set in Calibri and the rest of the manuscript in the Times New Roman)
