# Supplementary material for: From words to action? Linking ESG reports to environmental performance
Source: PLoS One. 2026 Jun 16;21(6):e0350762. doi: 10.1371/journal.pone.0350762 (PMC13271466; doi:10.1371/journal.pone.0350762)
Supplement: Attachment — Submitted filename: report.docx [file pone.0350762.s006.docx]

In introduction and literature review section, firstly, the theoretical framing can be strengthened to make the “words to action” argument sharper and more falsifiable. The manuscript cites greenwashing and talk action gaps, but the mechanisms that translate disclosure salience into operational change remain somewhat implicit. I suggest the authors more explicitly anchor the narrative in complementary disclosure theories such as legitimacy theory, impression management, and symbolic management, and then state clear, testable expectations for why certain environmental themes should correlate with KPI improvements while others might not. Secondly, the paper would benefit from a clearer positioning relative to the rapidly growing computational sustainability disclosure literature. The review currently covers several topic modeling and classification studies, but it should more precisely articulate what is methodologically new here, what is substantively new, and what prior findings this study corroborates or challenges, especially given the short KPI window and the Europe 600 sample scope.

In data and empirical research section, firstly, several design choices in the text pipeline merit deeper justification and additional robustness evidence. The decision to split reports into 10,000 word segments and then aggregate topic prevalence back to the report level is reasonable, but the manuscript should report more diagnostics showing that key topics and their prevalence are stable under alternative segmentation choices, alternative preprocessing, and alternative model specifications. The authors mention a 3,000 word threshold check, but readers will need to see which results remain stable, with a concise set of sensitivity tables or an online appendix summary. The translation of non English reports via DeepL also introduces a potential measurement error channel, so it would be important to add a robustness check restricting to English only reports, or at minimum to quantify the share of translated documents and assess whether translation is systematically associated with country or sector patterns. Secondly, the identification and inference strategy for linking topic prevalence to KPI changes needs tighter handling. Since KPI availability is concentrated in 2019 to 2023, reverse causality and contemporaneous reporting reactions are plausible. A more defensible specification would lag topic measures and explain next year KPI changes, or use multi year changes where feasible. The current setup also estimates multiple topic KPI relationships, so the manuscript should address multiple hypothesis testing, for example false discovery rate control, and ensure standard errors are clustered at the firm level given serial correlation. Finally, the authors omit tables and figures from PDF extraction, which may systematically remove KPI disclosure content or structured GRI style reporting that matters for environmental performance signals, so discussing the direction of this potential bias would improve credibility.

In discussion and implications section, firstly, the interpretation of “symbolic versus substantive” reporting should be made more cautious and evidence linked. The finding that renewable energy and sustainable value chain topics correlate with KPI improvements while emissions and electric vehicle topics do not is interesting, but alternative explanations exist, including sector compositional effects, baseline performance differences, and topic ambiguity. I recommend adding analyses that compare effect magnitudes across sectors, and that show whether topic shifts predict improvements more strongly among initially low performers, which would better support the claimed mechanism. In addition, the hierarchical clustering into environmental topic profiles is promising, yet it currently reads as descriptive. The manuscript should provide cluster validation metrics, stability checks, and a clearer interpretation of what each cluster implies for strategy, regulatory exposure, and operational capability. Secondly, policy and managerial implications would benefit from more operational specificity. If some themes appear more aligned with measurable environmental improvements, the paper can translate this into concrete guidance for regulators and assurance providers on which disclosure components are more informative, and for investors on how to interpret topic emphasis in ESG narratives. These recommendations should include a clear pathway, for example how standard setters might require more verifiable indicators for themes that show weak alignment with KPIs, and how firms might reduce boilerplate by linking narrative emphasis to audited performance metrics.

Overall recommendation: the paper has a clear question, a valuable dataset, and a promising text plus KPI linkage design, but it requires substantial strengthening in the identification logic, robustness reporting, and interpretive discipline before it can be considered for publication.
