# Supplementary material for: From words to action? Linking ESG reports to environmental performance
Source: PLoS One. 2026 Jun 16;21(6):e0350762. doi: 10.1371/journal.pone.0350762 (PMC13271466; doi:10.1371/journal.pone.0350762)
Supplement: Attachment — Submitted filename: Response to Reviewers Third revision.docx [file pone.0350762.s011.docx]

**Response to Reviewers**

Manuscript title: From Words to Action? Linking Sustainability Reports to Environmental Performance

Manuscript ID: PONE-D-25-44096R2

**Response to Reviewer #4**

General Information:

The new version of this paper is well-improved. The authors properly revised the paper by adding more convincing evidence. Here are some minor suggestions based on the latest version of the paper, which are listed as follows in order of appearance.

*We thank the reviewer for the positive assessment of the revised manuscript and for the additional comments. We have carefully revisited all points raised. However, we note that these issues were already addressed in detail in the previous round. For clarity, we briefly reiterate how each point has been incorporated in the manuscript.*

Minor Comments:

1. The literature review should be presented though in a clearer manner how the present study differentiates from existing research and makes a step towards better understanding the research problem. I recommend the authors to consult the following survey and empirical papers to contextualize your findings. This should help the readers to understand the novelty of your work (literature review section):

Kyriazis, N. A., Dimitriadis, K. A., & Theodossiou, P. (2026). Can Banking Intermediates Crowd Out Their High‐Tech Promising Successors? A Financial Stress Perspective. International Journal of Finance & Economics.

*This point has been addressed in earlier revisions by strengthening the positioning of the paper in the Introduction and Literature Review. In particular: the research gap and novelty are explicitly stated in the Introduction. The distinction from prior literature and the methodological contribution (linking STM to firm-level KPIs at the topic level) are clarified in Sections 1 and 2.4. The contribution is reiterated in the Conclusion.*

*Regarding the suggested reference (Kyriazis et al., 2026), we note that it is not directly relevant to the research question of our paper, which focuses on ESG reporting and environmental performance. We have therefore prioritised literature that is more closely aligned with the scope of our study.*

2. I want to see the innovation in this study from the previous studies, to see the difference between them. The research gap that is covered in this research article. Please incorporate these to be clear in the abstract, introduction and the conclusion of the paper.

*This point has already been addressed explicitly in the manuscript.*

- *In the Abstract, we state: “Methodologically, our study advances prior work by combining structural topic modelling with firm-level KPI analysis to evaluate ESG disclosure at the level of specific environmental themes rather than aggregate reporting measures.”*
- *In the Introduction, we define the research gap as follows: “Yet a central problem persists: it remains unclear whether the environmental issues companies emphasise in their ESG reports correspond to actual improvements in environmental performance.”*
- *Also in the Introduction, we formulate our contribution directly: “This study makes two contributions…Existing research has examined the volume, sentiment, or general presence of sustainability disclosures, but has not been able to assess whether the specific environmental issues firms emphasise correspond to measurable changes in environmental outcomes…Our study differs in two respects. Methodologically, we link unsupervised structural topic modelling directly to firm-level environmental KPIs at the level of specific environmental themes. Substantively, we show that the relationship between ESG communication and environmental performance is topic dependent.”*
- *In the Conclusion, we reiterate the novelty of the study: “These findings address our central research questions by revealing which topics are emphasised in ESG reports, how they differ across time and sectors, and—critically—how they correlate with measurable environmental outcomes. We thus provide the first large-scale empirical test of the alignment between ESG communication and real-world environmental performance.”*

*We therefore believe that the innovation, research gap, and differentiation from prior studies are already clearly stated in the relevant sections of the manuscript.*

3. How robust is the causal interpretation of the relationship between ESG topic emphasis and environmental KPIs, given that the analysis is based on contemporaneous associations rather than long-term dynamics?

*Our manuscript explicitly avoids causal claims, as stated in multiple sections:*

*In Section 3.2, we clarify the nature of the analysis:*

*“our analysis should be interpreted as evidence of contemporaneous alignment between reporting emphasis and environmental performance rather than long-term dynamics.”*

*In the Discussion and Conclusion, we explicitly state the correlational nature of the empirical design:*

*“A key limitation of our study is its correlational nature of its regression analysis… our analysis does not establish causal effects.”*

*We further clarify the implications:*

*“The links we identify therefore should not be interpreted as evidence that reporting choices cause improvements in environmental performance.”*

*These statements clearly establish that the analysis is correlational and that no causal interpretation is claimed.*

4. To what extent does the use of structural topic modelling (STM) accurately capture meaningful environmental themes, and how sensitive are the results to the chosen number of topics and preprocessing steps?

*This issue has already been addressed in both the manuscript and our earlier responses.*

*On preprocessing, Section 3.2 states: “All these preprocessing steps follow standard practice for bag-of-words topic models and are appropriate for STM […] Preprocessing steps such as stop-word removal, lemmatization, and bigram construction follow standard practice* *in bag-of-words topic modelling and are intended to enhance interpretability rather than to define alternative model specifications (40,45,53)”*

*In our earlier response, we clarified why these are not usually treated as separate robustness dimensions: “In the STM literature, such steps are treated as necessary normalization procedures rather than robustness dimensions to be systematically varied.” We clarify this further in the revised manuscript.*

*On the number of topics, Section 3.2 explains that the model was selected using standard STM diagnostics, while our earlier response makes the point more explicitly: “The selection of 34 topics is based on established STM model selection diagnostics: held-out likelihood (predictive performance), semantic coherence, and exclusivity…” and “This triangulated approach represents standard practice in STM applications, where topic-number robustness is assessed via diagnostic trade-offs rather than by reporting multiple arbitrary specifications.”*

*On robustness, the manuscript does include an additional check based on segmentation: “As an additional robustness check, we varied the document segmentation threshold from 10,000 to 3,000 words, obtaining qualitatively similar topic structures (see Appendix B).”*

*We therefore believe the validity of the STM specification, the role of preprocessing, and the logic of model selection have already been clearly addressed.*

5. How do the authors address potential measurement inconsistencies in environmental KPIs (e.g., Refinitiv scores vs firm-reported data) when linking ESG reporting content to actual environmental performance?

*This issue has been already explicitly addressed in the manuscript. In Section 3.2, we distinguish between different KPI sources:*

*“we distinguish between KPIs provided directly by the companies … and the KPIs assigned by Thomson Reuters/Refinitiv…”*

*In the Discussion section, we acknowledge measurement limitations:*

*“environmental KPIs may themselves be imperfect or lagged indicators of underlying environmental action, reflecting measurement constraints documented in recent ESG research.”*

*We also interpret these measures differently, as reflected in the results section:*

*“The first two columns report results for the indicators reported directly by the companies… while the latter three models analyse scores provided to the companies by ESG rating agency (in our case, Refinitiv).”*

*We further emphasize interpretative caution:*

*“KPI data may also vary in completeness or measurement precision, which can introduce noise into the associations we estimate”.*

*These points directly address potential inconsistencies by distinguishing KPI types and explicitly discussing their limitations.*

**Response to Reviewer #5**

*(our responses are in italics)*

*We thank the reviewer for the constructive comments and the positive engagement with our manuscript. We address the two remaining points below.*

First, the biggest remaining issue is identification. The current analysis still shows association, but it still cannot clearly show direction. In other words, the paper still cannot tell whether changes in topic prevalence lead to changes in environmental performance, or whether firms with changing environmental performance adjust their ESG reporting emphasis. The authors have improved the wording by using contemporaneous alignment, and this is better than before. But the empirical design itself is still limited. I suggest the authors make this limitation even more explicit and, if possible, add a lagged specification or a lead-lag test.

*We agree that the empirical design does not establish directionality. However, we would like to clarify that this feature of the study is explicitly framed as correlational throughout the manuscript. In the Discussion section, we explicitly acknowledge this limitation:*

*“A key limitation of our study is its correlational nature… our analysis cannot establish causal effects.”*

*and*

*“The links we identify therefore should not be interpreted as evidence that reporting choices cause improvements in environmental performance.”*

*Given this framing, the objective of the empirical strategy is to identify conditional correlations. Correlations, by definition, are not directional and do not establish the direction of influence between variables.*

*Regarding the suggestion of lead–lag specifications, we agree that such approaches can be informative. However, the relatively short time dimension of our panel (2019–2023 for KPI data) limits the scope for meaningful dynamic analysis. More importantly, lead–lag structures do not fully address reverse causality, as firms may adjust their ESG reporting in anticipation of expected changes in environmental performance or as part of the same strategic decision. In such cases, temporal ordering does not imply causal direction. We therefore acknowledge this as an important direction for future research and have clarified this point in the manuscript.*

Second, the statistical part is still not strong enough. The paper now explains the fixed effects setting and reports VIF results, which is useful. But the manuscript still needs to say more clearly how the standard errors are handled, especially whether they are clustered at the firm level. This is important because the analysis is based on panel data. Also, the paper tests several environmental topics and several environmental KPIs at the same time, so the multiple testing issue should be discussed more seriously. I suggest the authors clarify these points in the paper and provide stronger robustness on inference.

*We appreciate this important comment. In the revised version, we explicitly clarify the treatment of standard errors and strengthen inference by implementing Driscoll–Kraay standard errors, which account for heteroskedasticity serial correlation and cross-sectional dependence in panel data settings. This approach is more general than clustering at the firm level, as it additionally accounts for cross-sectional dependence across firms. We have added this clarification to the manuscript and updated the regression results accordingly. As you can see from the updated Table 4, our results remain robust. This strengthens the robustness of our inference while preserving the original findings.*

*As for your second point, the issue of multiple testing is mitigated by the fact that our analysis is structured around a limited and theoretically motivated set of environmental topics and KPIs, rather than an exploratory search over a large number of specifications. Moreover, inference is based on a consistent regression framework with fixed effects and Driscoll–Kraay standard errors, which provides robust estimates in the panel setting. We have clarified this point in the manuscript.*
