# Supplementary material for: From words to action? Linking ESG reports to environmental performance
Source: PLoS One. 2026 Jun 16;21(6):e0350762. doi: 10.1371/journal.pone.0350762 (PMC13271466; doi:10.1371/journal.pone.0350762)
Supplement: Peer review history

# Peer review: Decision Letter 0

**DOI:** 10.1371/journal.pone.0350762.r001

**Authors:** Hafiz M. Sohail

28 Oct 2025

PONE-D-25-44096

From Words to Action? Linking Sustainability Reports to Environmental Performance

PLOS ONE

Dear Dr. Savin,

Thank you for submitting your manuscript to PLOS ONE. After careful consideration, we have decided that your manuscript does not meet our criteria for publication and must therefore be rejected.

Specifically: The manuscript lacks clear framing and updated literature, provides weak justification for its methods, and overstates findings with limited interpretation and vague policy implications.

I am sorry that we cannot be more positive on this occasion, but hope that you appreciate the reasons for this decision.

Kind regards,

Hafiz M. Sohail, Phd

Academic Editor

PLOS ONE

[Note: HTML markup is below. Please do not edit.]

Reviewers' comments:

Reviewer's Responses to Questions

**Comments to the Author**

1. Is the manuscript technically sound, and do the data support the conclusions?

The manuscript must describe a technically sound piece of scientific research with data that supports the conclusions. Experiments must have been conducted rigorously, with appropriate controls, replication, and sample sizes. The conclusions must be drawn appropriately based on the data presented.

Reviewer #1: Yes

Reviewer #2: Yes

**********

2. Has the statistical analysis been performed appropriately and rigorously?

Reviewer #1: Yes

Reviewer #2: I Don't Know

**********

3. Have the authors made all data underlying the findings in their manuscript fully available?

The PLOS Data policy requires authors to make all data underlying the findings described in their manuscript fully available without restriction, with rare exception (please refer to the Data Availability Statement in the manuscript PDF file). The data should be provided as part of the manuscript or its supporting information, or deposited to a public repository. For example, in addition to summary statistics, the data points behind means, medians and variance measures should be available. If there are restrictions on publicly sharing data—e.g. participant privacy or use of data from a third party—those must be specified.

Reviewer #1: Yes

Reviewer #2: Yes

**********

4. Is the manuscript presented in an intelligible fashion and written in standard English?

PLOS ONE does not copyedit accepted manuscripts, so the language in submitted articles must be clear, correct, and unambiguous. Any typographical or grammatical errors should be corrected at revision, so please note any specific errors here.

Reviewer #1: Yes

Reviewer #2: Yes

**********

5. Review Comments to the Author

Please use the space provided to explain your answers to the questions above. You may also include additional comments for the author, including concerns about dual publication, research ethics, or publication ethics. (Please upload your review as an attachment if it exceeds 20,000 characters)

Reviewer #1: The manuscript addresses a critically important question in the ESG space with a robust methodological approach but to help strengthen it for publication, I have compiled my main comments below.

Abstract

1. Explicitly mention the geographic and regulatory context of the study to immediately frame the generalizability of the findings.

2. Clarify the nature of the findings by using more precise language and avoid causation.

Introduction:

1. Strengthen the opening by defining the research gap more sharply. “Little is known” is a weak choice of words and implies vagueness, articulate it with clarity.

2. Improve the logical flow between the problem of un-audited reports, the practice of greenwashing, and the proposed methodological solution (computational linguistics). The connection should be made more explicit.

3. Literature Review

1. Reorganize the literature review into clearly labeled sub-sections to enhance readability and structure.

2. Incorporate more recent literature (2021-2025) on greenwashing, the EU's Sustainable Finance Disclosure Regulation (SFDR), and the Corporate Sustainability Reporting Directive (CSRD) to ensure the review reflects the current, rapidly evolving regulatory and academic landscape.

4. Data and Methods

1. Provide a more detailed justification for the choice of Structural Topic Modeling (STM) over other methods like LDA. Explicitly state that the ability to incorporate metadata (sector, year) was a key factor, leading to more coherent and context-aware topics.

2. Offer a clearer rationale for the decision to split reports into 10,000-word chunks. Explain why this approach was necessary for topic quality and how the aggregation back to the report level preserves the integrity of the analysis for the subsequent regression.

3. Discuss potential limitations introduced by text pre-processing, such as the loss of nuanced meaning during lemmatization or the removal of tables/figures that might contain crucial environmental data.

4. In the regression analysis (Equation 2), provide a stronger theoretical and econometric justification for including both the level and the first difference of topic prevalence as independent variables. Acknowledge that this is a non-standard specification and defend its use.

5. Comment on the very low R-squared values in some regressions (e.g., Energy Intensity, ESG Score). Discuss what this implies for the model's explanatory power and the potential for other, unobserved factors driving KPI changes.

5. Results

1. Improve the interpretation of statistical results in the text. For each significant coefficient in Table 4, provide a clear, non-technical explanation of what it means (e.g., "A one-percentage-point increase in the prevalence of Topic X is associated with a change of Y in emission intensity").

2. In Table 2, consider adding a column for the number of reports in which each topic is a dominant or significant theme, to give readers a better sense of topic distribution across the corpus.

3. Ensure all figures referenced in the main text (Figures 1-7) are included and are of publication quality in the final submission.

6. Discussion

1. More explicitly state the novel contribution of the study in the opening of the discussion.

2. Temper the language regarding "greenwashing." The absence of a significant correlation is not definitive proof of greenwashing. Acknowledge alternative explanations, such as reporting on EVs or GHG that have not yet yielded measurable results.

3. Elaborate on the policy and practical implications. Provide specific, actionable recommendations.

7. Conclusion

1. Provide more specific and concrete suggestions for future research. Instead of "extending the analysis to other regions," suggest specific regions for comparison (e.g., "a comparative analysis between EU firms under CSRD and US firms under SEC proposed rules").

2. Recommend future studies to use more granular KPIs, such as Scope 3 emissions or water usage intensity, to create even stronger links with specific report topics.

3. Explicitly state the primary limitation of the study: the correlational nature of the analysis and the inability to make definitive causal claims about the relationship between reporting and performance.

8. Grammar and Writing Style

1. Maintain the coherence and flow throughout the manuscript. Simplify complex sentence structures, particularly in the Methods and Results sections. For example, break down long sentences explaining the STM process or regression outputs into shorter, more digestible statements.

2. Ensure consistent use of terminology (e.g., stick with "ESG reports" or "sustainability reports" rather than using them interchangeably without need).

3. Ensure the numbering (numeric values format) is consistent in manuscript.

Reviewer #2: Thank you for the opportunity to review this very interesting manuscript.

The topic is very current and the main claims are significant especially for the discipline of environmental communication and ethics, adequately placed in the context of preceding literature on this topic. The data analysis seems adequate for justifying the results.

However, there are few things that I noted:

- In the end of the section 3.2 the authors present two hypotheses (which should also include a null hypothesis?). A clear statement addressing whether to support or refute these two hypotheses should be more clearly made in the the Results -section. Did the authors use the methods to actually find answers to these particular hypotheses?

In the Conclusions -section the authors state that "These findings address our central research questions by revealing which topics are emphasized in ESG reports, how they differ across time and sectors, and—critically—

how they correlate with measurable environmental outcomes." (The presented hypotheses are not addressed at all).

- The Conclusions section was missing reflection on the Limitations of this study, which should be included in the manuscript. For instance, is there something that cannot be found out with the used methods, or are there risk of errors, biases etc. and where. Also, how adaptive is this method for, for instance, when new environmental KPIs are included or if there are environmental actions that are not communicated through ESG reports but that still may impact the environmental performance of the company?

- Also, I noted few minor typing mistakes in the manuscript. For example, in figure 7 the first cluster is titled as a Cluster 1 but rest of the clusters are titled as Groups.

Otherwise I thank you for your work and wish you good luck!

**********

6. PLOS authors have the option to publish the peer review history of their article (what does this mean?). If published, this will include your full peer review and any attached files.

If you choose “no”, your identity will remain anonymous but your review may still be made public.

**Do you want your identity to be public for this peer review?**  For information about this choice, including consent withdrawal, please see our Privacy Policy.

Reviewer #1: No

Reviewer #2: No

**********

[NOTE: If reviewer comments were submitted as an attachment file, they will be attached to this email and accessible via the submission site. Please log into your account, locate the manuscript record, and check for the action link "View Attachments". If this link does not appear, there are no attachment files.]

- - - - -

For journal use only: PONEDEC3

# Author comment: Author response to Decision Letter 1

**DOI:** 10.1371/journal.pone.0350762.r002

22 Dec 2025

We sincerely thank both reviewers for their thoughtful, constructive, and highly valuable feedback. Their comments provided clear guidance on how to sharpen the theoretical framing, strengthen the methodological transparency, and improve the clarity and relevance of the manuscript.

In response, we undertook substantial revisions across the Introduction, Literature Review, Data and Methods, Results, Discussion, and Conclusion sections. These include: a fully reorganized and significantly expanded literature review; a clearer articulation of the research gap and contribution; strengthened methodological explanations, including justifications for key analytic choices; more explicit links between hypotheses and results; a tempered and more nuanced interpretation of findings; and an extended discussion of policy and practical implications as well as study limitations.

We believe these revisions significantly enhance the clarity, rigor, and contribution of the manuscript. Below, we provide a point-by-point response to each comment.

Please find a detailed response to reviewers attached.

Attachment Submitted filename: 251212 Response to Reviewers FINAL.docx

# Peer review: Decision Letter 1

**DOI:** 10.1371/journal.pone.0350762.r003

**Authors:** Hafiz M. Sohail

3 Mar 2026

PONE-D-25-44096R1

From Words to Action? Linking ESG Reports to Environmental Performance

PLOS One

Dear Dr.Savin,

Thank you for submitting your manuscript to PLOS ONE. After careful consideration, we feel that it has merit but does not fully meet PLOS ONE’s publication criteria as it currently stands. Therefore, we invite you to submit a revised version of the manuscript that addresses the points raised during the review process.

Please submit your revised manuscript by April 1,2026 11:59pm. If you will need more time than this to complete your revisions, please reply to this message or contact the journal office at plosone@plos.org. When you're ready to submit your revision, log on to https://www.editorialmanager.com/pone/ and select the 'Submissions Needing Revision' folder to locate your manuscript file.

Please include the following items when submitting your revised manuscript:

- A letter that responds to each point raised by the academic editor and reviewer(s). You should upload this letter as a separate file labeled 'Response to Reviewers'.
- A marked-up copy of your manuscript that highlights changes made to the original version. You should upload this as a separate file labeled 'Revised Manuscript with Track Changes'.
- An unmarked version of your revised paper without tracked changes. You should upload this as a separate file labeled 'Manuscript'.

If you would like to make changes to your financial disclosure, please include your updated statement in your cover letter. Guidelines for resubmitting your figure files are available below the reviewer comments at the end of this letter.

If applicable, we recommend that you deposit your laboratory protocols in protocols.io to enhance the reproducibility of your results. Protocols.io assigns your protocol its own identifier (DOI) so that it can be cited independently in the future. For instructions see: https://journals.plos.org/plosone/s/submission-guidelines#loc-laboratory-protocols. Additionally, PLOS ONE offers an option for publishing peer-reviewed Lab Protocol articles, which describe protocols hosted on protocols.io. Read more information on sharing protocols at https://plos.org/protocols?utm_medium=editorial-email&utm_source=authorletters&utm_campaign=protocols.

We look forward to receiving your revised manuscript.

Kind regards,

Hafiz M. Sohail, Phd

Academic Editor

PLOS One

Journal Requirements:

When submitting your revision, we need you to address these additional requirements.

1.Please ensure that your manuscript meets PLOS ONE's style requirements, including those for file naming. The PLOS ONE style templates can be found at

https://journals.plos.org/plosone/s/file?id=wjVg/PLOSOne_formatting_sample_main_body.pdf and

https://journals.plos.org/plosone/s/file?id=ba62/PLOSOne_formatting_sample_title_authors_affiliations.pdf

2. In the online submission form, you indicated that "The data underlying the results presented in the study are available from authors on request".

All PLOS journals now require all data underlying the findings described in their manuscript to be freely available to other researchers, either 1. In a public repository, 2. Within the manuscript itself, or 3. Uploaded as supplementary information.

This policy applies to all data except where public deposition would breach compliance with the protocol approved by your research ethics board. If your data cannot be made publicly available for ethical or legal reasons (e.g., public availability would compromise patient privacy), please explain your reasons on resubmission and your exemption request will be escalated for approval.

3. PLOS requires an ORCID iD for the corresponding author in Editorial Manager on papers submitted after December 6th, 2016. Please ensure that you have an ORCID iD and that it is validated in Editorial Manager. To do this, go to ‘Update my Information’ (in the upper left-hand corner of the main menu), and click on the Fetch/Validate link next to the ORCID field. This will take you to the ORCID site and allow you to create a new iD or authenticate a pre-existing iD in Editorial Manager.

4. We note that Figure 1 in your submission contain map images which may be copyrighted. All PLOS content is published under the Creative Commons Attribution License (CC BY 4.0), which means that the manuscript, images, and Supporting Information files will be freely available online, and any third party is permitted to access, download, copy, distribute, and use these materials in any way, even commercially, with proper attribution. For these reasons, we cannot publish previously copyrighted maps or satellite images created using proprietary data, such as Google software (Google Maps, Street View, and Earth). For more information, see our copyright guidelines: http://journals.plos.org/plosone/s/licenses-and-copyright.

We require you to either (1) present written permission from the copyright holder to publish these figures specifically under the CC BY 4.0 license, or (2) remove the figures from your submission:

1. You may seek permission from the original copyright holder of Figure 1 to publish the content specifically under the CC BY 4.0 license.

We recommend that you contact the original copyright holder with the Content Permission Form (http://journals.plos.org/plosone/s/file?id=7c09/content-permission-form.pdf) and the following text:

“I request permission for the open-access journal PLOS ONE to publish XXX under the Creative Commons Attribution License (CCAL) CC BY 4.0 (http://creativecommons.org/licenses/by/4.0/). Please be aware that this license allows unrestricted use and distribution, even commercially, by third parties. Please reply and provide explicit written permission to publish XXX under a CC BY license and complete the attached form.”

Please upload the completed Content Permission Form or other proof of granted permissions as an "Other" file with your submission.

In the figure caption of the copyrighted figure, please include the following text: “Reprinted from [ref] under a CC BY license, with permission from [name of publisher], original copyright [original copyright year].”

2. If you are unable to obtain permission from the original copyright holder to publish these figures under the CC BY 4.0 license or if the copyright holder’s requirements are incompatible with the CC BY 4.0 license, please either i) remove the figure or ii) supply a replacement figure that complies with the CC BY 4.0 license. Please check copyright information on all replacement figures and update the figure caption with source information. If applicable, please specify in the figure caption text when a figure is similar but not identical to the original image and is therefore for illustrative purposes only.

The following resources for replacing copyrighted map figures may be helpful:

USGS National Map Viewer (public domain): http://viewer.nationalmap.gov/viewer/

The Gateway to Astronaut Photography of Earth (public domain): http://eol.jsc.nasa.gov/sseop/clickmap/

Maps at the CIA (public domain): https://www.cia.gov/library/publications/the-world-factbook/index.html and https://www.cia.gov/library/publications/cia-maps-publications/index.html

NASA Earth Observatory (public domain): http://earthobservatory.nasa.gov/

Landsat: http://landsat.visibleearth.nasa.gov/

USGS EROS (Earth Resources Observatory and Science (EROS) Center) (public domain): http://eros.usgs.gov/#

Natural Earth (public domain): http://www.naturalearthdata.com/

5. Please remove your figures from within your manuscript file, leaving only the individual TIFF/EPS image files, uploaded separately. These will be automatically included in the reviewers’ PDF.

If the reviewer comments include a recommendation to cite specific previously published works, please review and evaluate these publications to determine whether they are relevant and should be cited. There is no requirement to cite these works unless the editor has indicated otherwise.

Additional Editor Comments (if provided):

[Note: HTML markup is below. Please do not edit.]

Reviewers' comments:

Reviewer's Responses to Questions

**Comments to the Author**

1. If the authors have adequately addressed your comments raised in a previous round of review and you feel that this manuscript is now acceptable for publication, you may indicate that here to bypass the “Comments to the Author” section, enter your conflict of interest statement in the “Confidential to Editor” section, and submit your "Accept" recommendation.

Reviewer #3: (No Response)

Reviewer #4: (No Response)

Reviewer #5: (No Response)

**********

2. Is the manuscript technically sound, and do the data support the conclusions?

The manuscript must describe a technically sound piece of scientific research with data that supports the conclusions. Experiments must have been conducted rigorously, with appropriate controls, replication, and sample sizes. The conclusions must be drawn appropriately based on the data presented.

Reviewer #3: Yes

Reviewer #4: Yes

Reviewer #5: Partly

**********

3. Has the statistical analysis been performed appropriately and rigorously?

Reviewer #3: Yes

Reviewer #4: Yes

Reviewer #5: Yes

**********

4. Have the authors made all data underlying the findings in their manuscript fully available?

The PLOS Data policy requires authors to make all data underlying the findings described in their manuscript fully available without restriction, with rare exception (please refer to the Data Availability Statement in the manuscript PDF file). The data should be provided as part of the manuscript or its supporting information, or deposited to a public repository. For example, in addition to summary statistics, the data points behind means, medians and variance measures should be available. If there are restrictions on publicly sharing data—e.g. participant privacy or use of data from a third party—those must be specified.

Reviewer #3: Yes

Reviewer #4: No

Reviewer #5: No

**********

5. Is the manuscript presented in an intelligible fashion and written in standard English?

PLOS ONE does not copyedit accepted manuscripts, so the language in submitted articles must be clear, correct, and unambiguous. Any typographical or grammatical errors should be corrected at revision, so please note any specific errors here.

Reviewer #3: Yes

Reviewer #4: Yes

Reviewer #5: Yes

**********

6. Review Comments to the Author

Please use the space provided to explain your answers to the questions above. You may also include additional comments for the author, including concerns about dual publication, research ethics, or publication ethics. (Please upload your review as an attachment if it exceeds 20,000 characters)

Reviewer #3: Title and Abstract

1. Consider slightly rephrasing the abstract to emphasize the methodological novelty (topic modeling + KPI linkage) upfront.

Data and Methods

2. The handling of long reports by splitting into 10,000-word segments is reasonable but should include a brief robustness note in the main text (not just the appendix).

3. The regression specification (Equation 2) is sound, but please clarify why both level and change in topic prevalence are included in the same model—consider a brief note on multicollinearity checks.

Results

4. In Table 4, some coefficient signs are counterintuitive (e.g., T8 Sustainable VCs positive for emission intensity). Please provide a brief interpretation in text.

5. The discussion of why certain topics (e.g., GHG emissions, electric vehicles) show no significant KPI association is appropriately cautious and nuanced.

References and Supplementary Material

6. Appendix tables and figures are helpful; ensure all are referenced in the main text.

Minor Editorial Issues

7. Some formatting inconsistencies remain (e.g., “organisation” vs. “organization,” figure callouts like “Figure 4.4.”). Please standardize.

8. A few typos/grammatical errors persist (e.g., p. 84 “sizesise” → “size”). A final proofread is recommended.

9. All text should be in the same font style (headings are set in Calibri and the rest of the manuscript in the Times New Roman)

Reviewer #4: I am attaching my review report in PDF file format for your consideration. The document contains my detailed assessment of the manuscript, including comments on its theoretical contribution, methodology, empirical analysis, and overall clarity of presentation.

Reviewer #5: In introduction and literature review section, firstly, the theoretical framing can be strengthened to make the “words to action” argument sharper and more falsifiable. The manuscript cites greenwashing and talk action gaps, but the mechanisms that translate disclosure salience into operational change remain somewhat implicit. I suggest the authors more explicitly anchor the narrative in complementary disclosure theories such as legitimacy theory, impression management, and symbolic management, and then state clear, testable expectations for why certain environmental themes should correlate with KPI improvements while others might not. Secondly, the paper would benefit from a clearer positioning relative to the rapidly growing computational sustainability disclosure literature. The review currently covers several topic modeling and classification studies, but it should more precisely articulate what is methodologically new here, what is substantively new, and what prior findings this study corroborates or challenges, especially given the short KPI window and the Europe 600 sample scope.

In data and empirical research section, firstly, several design choices in the text pipeline merit deeper justification and additional robustness evidence. The decision to split reports into 10,000 word segments and then aggregate topic prevalence back to the report level is reasonable, but the manuscript should report more diagnostics showing that key topics and their prevalence are stable under alternative segmentation choices, alternative preprocessing, and alternative model specifications. The authors mention a 3,000 word threshold check, but readers will need to see which results remain stable, with a concise set of sensitivity tables or an online appendix summary. The translation of non English reports via DeepL also introduces a potential measurement error channel, so it would be important to add a robustness check restricting to English only reports, or at minimum to quantify the share of translated documents and assess whether translation is systematically associated with country or sector patterns. Secondly, the identification and inference strategy for linking topic prevalence to KPI changes needs tighter handling. Since KPI availability is concentrated in 2019 to 2023, reverse causality and contemporaneous reporting reactions are plausible. A more defensible specification would lag topic measures and explain next year KPI changes, or use multi year changes where feasible. The current setup also estimates multiple topic KPI relationships, so the manuscript should address multiple hypothesis testing, for example false discovery rate control, and ensure standard errors are clustered at the firm level given serial correlation. Finally, the authors omit tables and figures from PDF extraction, which may systematically remove KPI disclosure content or structured GRI style reporting that matters for environmental performance signals, so discussing the direction of this potential bias would improve credibility.

In discussion and implications section, firstly, the interpretation of “symbolic versus substantive” reporting should be made more cautious and evidence linked. The finding that renewable energy and sustainable value chain topics correlate with KPI improvements while emissions and electric vehicle topics do not is interesting, but alternative explanations exist, including sector compositional effects, baseline performance differences, and topic ambiguity. I recommend adding analyses that compare effect magnitudes across sectors, and that show whether topic shifts predict improvements more strongly among initially low performers, which would better support the claimed mechanism. In addition, the hierarchical clustering into environmental topic profiles is promising, yet it currently reads as descriptive. The manuscript should provide cluster validation metrics, stability checks, and a clearer interpretation of what each cluster implies for strategy, regulatory exposure, and operational capability. Secondly, policy and managerial implications would benefit from more operational specificity. If some themes appear more aligned with measurable environmental improvements, the paper can translate this into concrete guidance for regulators and assurance providers on which disclosure components are more informative, and for investors on how to interpret topic emphasis in ESG narratives. These recommendations should include a clear pathway, for example how standard setters might require more verifiable indicators for themes that show weak alignment with KPIs, and how firms might reduce boilerplate by linking narrative emphasis to audited performance metrics.

Overall recommendation: the paper has a clear question, a valuable dataset, and a promising text plus KPI linkage design, but it requires substantial strengthening in the identification logic, robustness reporting, and interpretive discipline before it can be considered for publication.

**********

7. PLOS authors have the option to publish the peer review history of their article (what does this mean?). If published, this will include your full peer review and any attached files.

If you choose “no”, your identity will remain anonymous but your review may still be made public.

**Do you want your identity to be public for this peer review?**  For information about this choice, including consent withdrawal, please see our Privacy Policy.

Reviewer #3: No

Reviewer #4: No

Reviewer #5: **Yes:** Jinyu Chen

**********

[NOTE: If reviewer comments were submitted as an attachment file, they will be attached to this email and accessible via the submission site. Please log into your account, locate the manuscript record, and check for the action link "View Attachments". If this link does not appear, there are no attachment files.]

To ensure your figures meet our technical requirements, please review our figure guidelines: https://journals.plos.org/plosone/s/figures

You may also use PLOS’s free figure tool, NAAS, to help you prepare publication quality figures: https://journals.plos.org/plosone/s/figures#loc-tools-for-figure-preparation.

NAAS will assess whether your figures meet our technical requirements by comparing each figure against our figure specifications.

Attachment Submitted filename: PONE-D-25-44096_R1 Overall Recommendation.docx

Attachment Submitted filename: Review on manuscript ESG Reports .pdf

Attachment Submitted filename: report.docx

# Author comment: Author response to Decision Letter 2

**DOI:** 10.1371/journal.pone.0350762.r004

18 Mar 2026

the detailed response to reviewers is atatched to the revision

# Peer review: Decision Letter 2

**DOI:** 10.1371/journal.pone.0350762.r005

**Authors:** Hafiz M. Sohail

9 Apr 2026

PONE-D-25-44096R2

From Words to Action? Linking ESG Reports to Environmental Performance

PLOS One

Dear Dr.  Savin,

Thank you for submitting your manuscript to PLOS ONE. After careful consideration, we feel that it has merit but does not fully meet PLOS ONE’s publication criteria as it currently stands. Therefore, we invite you to submit a revised version of the manuscript that addresses the points raised during the review process.

Please submit your revised manuscript by May 24 2026 11:59PM. If you will need more time than this to complete your revisions, please reply to this message or contact the journal office at plosone@plos.org. When you're ready to submit your revision, log on to https://www.editorialmanager.com/pone/ and select the 'Submissions Needing Revision' folder to locate your manuscript file.

Please include the following items when submitting your revised manuscript:

- A letter that responds to each point raised by the academic editor and reviewer(s). You should upload this letter as a separate file labeled 'Response to Reviewers'.
- A marked-up copy of your manuscript that highlights changes made to the original version. You should upload this as a separate file labeled 'Revised Manuscript with Track Changes'.
- An unmarked version of your revised paper without tracked changes. You should upload this as a separate file labeled 'Manuscript'.

If you would like to make changes to your financial disclosure, please include your updated statement in your cover letter. Guidelines for resubmitting your figure files are available below the reviewer comments at the end of this letter.

If applicable, we recommend that you deposit your laboratory protocols in protocols.io to enhance the reproducibility of your results. Protocols.io assigns your protocol its own identifier (DOI) so that it can be cited independently in the future. For instructions see: https://journals.plos.org/plosone/s/submission-guidelines#loc-laboratory-protocols. Additionally, PLOS ONE offers an option for publishing peer-reviewed Lab Protocol articles, which describe protocols hosted on protocols.io. Read more information on sharing protocols at https://plos.org/protocols?utm_medium=editorial-email&utm_source=authorletters&utm_campaign=protocols.

As the corresponding author, your ORCID iD is verified in the submission system and will appear in the published article. PLOS supports the use of ORCID, and we encourage all coauthors to register for an ORCID iD and use it as well. Please encourage your coauthors to verify their ORCID iD within the submission system before final acceptance, as unverified ORCID iDs will not appear in the published article. *Only*  the individual author can complete the verification step; PLOS staff *cannot*  verify ORCID iDs on behalf of authors.

We look forward to receiving your revised manuscript.

Kind regards,

Hafiz M. Sohail, Phd

Academic Editor

PLOS One

Journal Requirements:

1. If the reviewer comments include a recommendation to cite specific previously published works, please review and evaluate these publications to determine whether they are relevant and should be cited. There is no requirement to cite these works unless the editor has indicated otherwise.

2. Please review your reference list to ensure that it is complete and correct. If you have cited papers that have been retracted, please include the rationale for doing so in the manuscript text, or remove these references and replace them with relevant current references. Any changes to the reference list should be mentioned in the rebuttal letter that accompanies your revised manuscript. If you need to cite a retracted article, indicate the article’s retracted status in the References list and also include a citation and full reference for the retraction notice.

[Note: HTML markup is below. Please do not edit.]

Reviewer's Responses to Questions

**Comments to the Author**

1. If the authors have adequately addressed your comments raised in a previous round of review and you feel that this manuscript is now acceptable for publication, you may indicate that here to bypass the “Comments to the Author” section, enter your conflict of interest statement in the “Confidential to Editor” section, and submit your "Accept" recommendation.

Reviewer #3: (No Response)

Reviewer #4: (No Response)

Reviewer #5: (No Response)

**********

2. Is the manuscript technically sound, and do the data support the conclusions?

The manuscript must describe a technically sound piece of scientific research with data that supports the conclusions. Experiments must have been conducted rigorously, with appropriate controls, replication, and sample sizes. The conclusions must be drawn appropriately based on the data presented.

Reviewer #3: (No Response)

Reviewer #4: Yes

Reviewer #5: Partly

**********

3. Has the statistical analysis been performed appropriately and rigorously?

Reviewer #3: (No Response)

Reviewer #4: Yes

Reviewer #5: Yes

**********

4. Have the authors made all data underlying the findings in their manuscript fully available?

The PLOS Data policy requires authors to make all data underlying the findings described in their manuscript fully available without restriction, with rare exception (please refer to the Data Availability Statement in the manuscript PDF file). The data should be provided as part of the manuscript or its supporting information, or deposited to a public repository. For example, in addition to summary statistics, the data points behind means, medians and variance measures should be available. If there are restrictions on publicly sharing data—e.g. participant privacy or use of data from a third party—those must be specified.

Reviewer #3: (No Response)

Reviewer #4: No

Reviewer #5: Yes

**********

5. Is the manuscript presented in an intelligible fashion and written in standard English?

PLOS ONE does not copyedit accepted manuscripts, so the language in submitted articles must be clear, correct, and unambiguous. Any typographical or grammatical errors should be corrected at revision, so please note any specific errors here.

Reviewer #3: (No Response)

Reviewer #4: Yes

Reviewer #5: Yes

**********

6. Review Comments to the Author

Please use the space provided to explain your answers to the questions above. You may also include additional comments for the author, including concerns about dual publication, research ethics, or publication ethics. (Please upload your review as an attachment if it exceeds 20,000 characters)

Reviewer #3: (No Response)

Reviewer #4: I am attaching my review report in PDF format for your consideration. The document contains my detailed assessment of the manuscript, including comments on its theoretical contribution, methodology, empirical analysis, and overall clarity of presentation.

Reviewer #5: First, the biggest remaining issue is identification. The current analysis still shows association, but it still cannot clearly show direction. In other words, the paper still cannot tell whether changes in topic prevalence lead to changes in environmental performance, or whether firms with changing environmental performance adjust their ESG reporting emphasis. The authors have improved the wording by using contemporaneous alignment, and this is better than before. But the empirical design itself is still limited. I suggest the authors make this limitation even more explicit and, if possible, add a lagged specification or a lead-lag test.

Second, the statistical part is still not strong enough. The paper now explains the fixed effects setting and reports VIF results, which is useful. But the manuscript still needs to say more clearly how the standard errors are handled, especially whether they are clustered at the firm level. This is important because the analysis is based on panel data. Also, the paper tests several environmental topics and several environmental KPIs at the same time, so the multiple testing issue should be discussed more seriously. I suggest the authors clarify these points in the paper and provide stronger robustness on inference.

**********

7. PLOS authors have the option to publish the peer review history of their article (what does this mean?). If published, this will include your full peer review and any attached files.

If you choose “no”, your identity will remain anonymous but your review may still be made public.

**Do you want your identity to be public for this peer review?**  For information about this choice, including consent withdrawal, please see our Privacy Policy.

Reviewer #3: No

Reviewer #4: No

Reviewer #5: **Yes:** Jinyu Chen

**********

[NOTE: If reviewer comments were submitted as an attachment file, they will be attached to this email and accessible via the submission site. Please log into your account, locate the manuscript record, and check for the action link "View Attachments". If this link does not appear, there are no attachment files.]

To ensure your figures meet our technical requirements, please review our figure guidelines: https://journals.plos.org/plosone/s/figures

You may also use PLOS’s free figure tool, NAAS, to help you prepare publication quality figures: https://journals.plos.org/plosone/s/figures#loc-tools-for-figure-preparation.

NAAS will assess whether your figures meet our technical requirements by comparing each figure against our figure specifications.

Attachment Submitted filename: Review 2 on manuscript Words to Action.pdf

Attachment Submitted filename: report.docx

# Author comment: Author response to Decision Letter 3

**DOI:** 10.1371/journal.pone.0350762.r006

23 Apr 2026

see the attached detailed point by point response attached

Attachment Submitted filename: Response to Reviewers Third revision.docx

# Peer review: Decision Letter 3

**DOI:** 10.1371/journal.pone.0350762.r007

**Authors:** Hafiz M. Sohail

18 May 2026

From Words to Action? Linking ESG Reports to Environmental Performance

PONE-D-25-44096R3

Dear Dr. Savin,

We’re pleased to inform you that your manuscript has been judged scientifically suitable for publication and will be formally accepted for publication once it meets all outstanding technical requirements.

Within one week, you’ll receive an e-mail detailing the required amendments. When these have been addressed, you’ll receive a formal acceptance letter and your manuscript will be scheduled for publication.

An invoice will be generated when your article is formally accepted. Please note, if your institution has a publishing partnership with PLOS and your article meets the relevant criteria, all or part of your publication costs will be covered. Please make sure your user information is up-to-date by logging into Editorial Manager at Editorial Manager® and clicking the ‘Update My Information' link at the top of the page. For questions related to billing, please contact billing support.

If your institution or institutions have a press office, please notify them about your upcoming paper to help maximize its impact. If they’ll be preparing press materials, please inform our press team as soon as possible -- no later than 48 hours after receiving the formal acceptance. Your manuscript will remain under strict press embargo until 2 pm Eastern Time on the date of publication. For more information, please contact onepress@plos.org.

Kind regards,

Hafiz M. Sohail, Phd

Academic Editor

PLOS One

Additional Editor Comments (optional):

Reviewers' comments:

Reviewer's Responses to Questions

**Comments to the Author**

1. If the authors have adequately addressed your comments raised in a previous round of review and you feel that this manuscript is now acceptable for publication, you may indicate that here to bypass the “Comments to the Author” section, enter your conflict of interest statement in the “Confidential to Editor” section, and submit your "Accept" recommendation.

Reviewer #4: All comments have been addressed

Reviewer #5: All comments have been addressed

**********

2. Is the manuscript technically sound, and do the data support the conclusions?

The manuscript must describe a technically sound piece of scientific research with data that supports the conclusions. Experiments must have been conducted rigorously, with appropriate controls, replication, and sample sizes. The conclusions must be drawn appropriately based on the data presented.

Reviewer #4: No

Reviewer #5: Yes

**********

3. Has the statistical analysis been performed appropriately and rigorously?

Reviewer #4: Yes

Reviewer #5: Yes

**********

4. Have the authors made all data underlying the findings in their manuscript fully available?

The PLOS Data policy requires authors to make all data underlying the findings described in their manuscript fully available without restriction, with rare exception (please refer to the Data Availability Statement in the manuscript PDF file). The data should be provided as part of the manuscript or its supporting information, or deposited to a public repository. For example, in addition to summary statistics, the data points behind means, medians and variance measures should be available. If there are restrictions on publicly sharing data—e.g. participant privacy or use of data from a third party—those must be specified.

Reviewer #4: Yes

Reviewer #5: Yes

**********

5. Is the manuscript presented in an intelligible fashion and written in standard English?

PLOS ONE does not copyedit accepted manuscripts, so the language in submitted articles must be clear, correct, and unambiguous. Any typographical or grammatical errors should be corrected at revision, so please note any specific errors here.

Reviewer #4: Yes

Reviewer #5: Yes

**********

6. Review Comments to the Author

Please use the space provided to explain your answers to the questions above. You may also include additional comments for the author, including concerns about dual publication, research ethics, or publication ethics. (Please upload your review as an attachment if it exceeds 20,000 characters)

Reviewer #4: (No Response)

Reviewer #5: (No Response)

**********

7. PLOS authors have the option to publish the peer review history of their article (what does this mean?). If published, this will include your full peer review and any attached files.

If you choose “no”, your identity will remain anonymous but your review may still be made public.

**Do you want your identity to be public for this peer review?**  For information about this choice, including consent withdrawal, please see our Privacy Policy.

Reviewer #4: No

Reviewer #5: **Yes:** Chen Jinyu

**********

# Editor report: Acceptance letter

**DOI:** 10.1371/journal.pone.0350762.r008

**Authors:** Hafiz M. Sohail

PONE-D-25-44096R3

PLOS One

Dear Dr. Savin,

I'm pleased to inform you that your manuscript has been deemed suitable for publication in PLOS One. Congratulations! Your manuscript is now being handed over to our production team.

At this stage, our production department will prepare your paper for publication. This includes ensuring the following:

* All references, tables, and figures are properly cited

* All relevant supporting information is included in the manuscript submission,

* There are no issues that prevent the paper from being properly typeset

You will receive further instructions from the production team, including instructions on how to review your proof when it is ready. Please keep in mind that we are working through a large volume of accepted articles, so please give us a few days to review your paper and let you know the next and final steps.

Lastly, if your institution or institutions have a press office, please let them know about your upcoming paper now to help maximize its impact. If they'll be preparing press materials, please inform our press team within the next 48 hours. Your manuscript will remain under strict press embargo until 2 pm Eastern Time on the date of publication. For more information, please contact onepress@plos.org.

You will receive an invoice from PLOS for your publication fee after your manuscript has reached the completed accept phase. If you receive an email requesting payment before acceptance or for any other service, this may be a phishing scheme. Learn how to identify phishing emails and protect your accounts at https://explore.plos.org/phishing.

If we can help with anything else, please email us at customercare@plos.org.

Thank you for submitting your work to PLOS ONE and supporting open access.

Kind regards,

PLOS ONE Editorial Office Staff

on behalf of

Dr. Hafiz M. Sohail

Academic Editor

PLOS One
